# CAMTA 1 regulates drought responses in *Arabidopsis thaliana*

**DOI:** 10.1186/1471-2164-14-216

**Published:** 2013-04-02

**Authors:** Neha Pandey, Alok Ranjan, Poonam Pant, Rajiv K Tripathi, Farha Ateek, Haushilla P Pandey, Uday V Patre, Samir V Sawant

**Affiliations:** 1 Council of Scientific and Industrial Research-National Botanical Research Institute, Rana Pratap Marg, Lucknow 226001, INDIA; 2 Present address: Centre for AIDS Health Disparities Research, Meharry Medical College, Nashville, TN 37208, USA; 3 Department of Biochemistry, Banaras Hindu University, Varanasi, India

**Keywords:** CAMTA1 mutant, WUE, RWC, Osmotic stress, Microarray, Gene expression, Drought recovery

## Abstract

**Background:**

Transcription factors (TF) play a crucial role in regulating gene expression and are fit to regulate diverse cellular processes by interacting with other proteins. A TF named calmodulin binding transcription activator (CAMTA) was identified in *Arabidopsis thaliana* (AtCAMTA1-6). To explore the role of CAMTA1 in drought response*,* the phenotypic differences and gene expression was studied between *camta1* and Col-0 under drought condition.

**Results:**

In *camta1,* root development was abolished showing high-susceptibility to induced osmotic stress resulting in small wrinkled rosette leaves and stunted primary root. In *camta1* under drought condition, we identified growth retardation, poor WUE, low photosystem II efficiency, decline in RWC and higher sensitivity to drought with reduced survivability. The microarray analysis of drought treated *camta1* revealed that CAMTA1 regulates “drought recovery” as most indicative pathway along with other stress response, osmotic balance, apoptosis, DNA methylation and photosynthesis. Interestingly, majority of positively regulated genes were related to plasma membrane and chloroplast. Further, our analysis indicates that CAMTA1 regulates several stress responsive genes including RD26, ERD7, RAB18, LTPs, COR78, CBF1, HSPs etc. and promoter of these genes were enriched with CAMTA recognition *cis*-element. CAMTA1 probably regulate drought recovery by regulating expression of AP2-EREBP transcription factors and Abscisic acid response.

**Conclusion:**

CAMTA1 rapidly changes broad spectrum of responsive genes of membrane integrity and photosynthetic machinery by generating ABA response for challenging drought stress. Our results demonstrate the important role of CAMTA1 in regulating drought response in *Arabidopsis,* thus could be genetically engineered for improving drought tolerance in crop.

## Background

Abiotic and biotic stress is one of the major environmental factors limiting crop productivity worldwide. Water deficiency is one of the primary causes for the reduction in crop yield [1]. Previously, Several studies shows that calcium, a key messenger, involved in several signalling pathways and regulates many growth and developmental processes, plays a crucial role in stress signalling and adaptation [2-6] and in response to various biotic (pathogens, defence elicitors, and insect feeding) and abiotic stresses such as light, UV light, high and low temperature, salt, drought, osmotic stress, mechanical stimuli including touch and wind, oxidative stress, ozone, and hypoxia [7-9]. Within cells, calcium signatures are perceived by the EF-hand families of calcium-modulated proteins (calmodulin - CaM, Calcium dependent protein kinase - CDPK and calcineurin B-like protein - CBL) which are well characterised in plants [10-12]. When intracellular calcium rises to about 1 μM, calmodulin (CaM) binds calcium, undergoes a change in conformation, and activates the target gene thereby producing the respective cellular response [4,6]. To elucidate the mechanisms underlying calcium/calmodulin regulated gene expression in plants, previous reports identified a family of six Arabidopsis genes encoding calmodulin binding transcription activators (CAMTAs) [5,13] also referred to as signal-responsive (SR) protein [14] or ethylene-induced CaM binding proteins (EICBP) [15]. This factor, designated AtCAMTA (*Arabidopsis thaliana* CaM-binding transcription activator), is highly conserved and contains a CG-1 homology DNA-binding domain at the N terminus (binding site includes the CGCG and CGTG motif), a TIG domain (an immunoglobulin-like fold involved in nonspecific DNA binding), three ankyrin repeats (implicated in protein-protein interaction) and five putative CaM-binding motifs called as IQ motif [5,13,14,16]. In Arabidopsis, there are six CAMTAs (CAMTA1-6), whose transcript accumulates (up-regulated) or diminish (down-regulated) rapidly and transiently to various abiotic and biotic stress. Each member has distinct or overlapping spatial and temporal expression pattern in different plant developmental stages under various biotic and abiotic stresses [15,17]. The first evidence of biological and physiological function of CAMTA protein was recently reported in Arabidopsis CAMTA3 (AtSR1) loss of function mutant through a reverse genetic approach [18]. CAMTA 3- knockout plants during developmental stages accumulates high level of reactive oxygen species (ROS), showed enhanced resistance towards fungal and bacterial pathogen by suppressing plant responses. It negatively regulates the defence response to pathogens and interacts with WRKY33 TF in *camta3* mutants [16,18]. Similarly another study by Galon Y. et al., 2010 on CAMTA1 reports the increased sensitivity for auxin in *camta1* mutant suggesting a role in suppressing the plant responses to auxin when induced under stress condition [19]. There is considerable information about the changes in gene expression regulated by CAMTA 1 under various stresses like cold, salt, heat and ultra-voilet [20,21]. The promoters of Drought responsive element binding protein 1C (DREB1C) and ZAT12 binds with CAMTA3 in plants [21] indicating a calcium-signal driven gene expression. Besides various findings on function of CAMTA protein on stress physiology, [22] were first to report down-stream gene of the CAMTA protein and showed pollen-specific expression of AtCAMTA1 and AtCAMTA5 which possibly increased *Arabidopsis* VPPase (AVP1) gene expression in pollen by binding to the pollen-specific *cis*-acting region of *AVP1 .* The DNA *cis-*element that binds to CAMTA was identified as CGCG and CGTG binding motif in Arabidopsis, AtCAMTA3 [14] and Rice, Os-CBT [23]. The consensus sequence of CGCG core motif is (A/C)**CGCG**(C/G/T), giving the name to the DNA binding domain of the protein as CG-1, a novel *cis*-element which was first isolated from the parsley cDNA library [24]. The consensus sequence of CGTG core motif is (A/C)**CGTG**T and includes classical abscisic acid responsive element (ABRE) motif (ACGTGT), which is recognised by bZIP proteins [25]. The most recent report on CAMTA (*SlSR)* in tomato revealed its role in fruit development and ripening [26], they cloned seven *SlSR* genes and their expression levels were differentially regulated mainly by development signals, as well as by ethylene and suggested that *SlSR*s were located downstream of the *Rin*-regulated network. On taking these results together, CAMTA have the potential of relaying calcium signalling via calmodulin binding domain and stress signalling via CG-1 and ABRE binding motif.

The aim of our research is to study and characterise the molecular function of CAMTA1 gene under drought condition and establish a possible role of CAMTA1 protein in drought stress. The present study provides considerable information about the changes in gene expression, metabolic pathways, CGTG and CGCG motif dependent gene expression in CAMTA1 under drought stress. In brief, we hypothesized CAMTA1 to be essential for successful drought recovery.

## Results

### The knockout *camta1* showed drought sensitivity, poor WUE and decline in RWC

**Figure 1:**
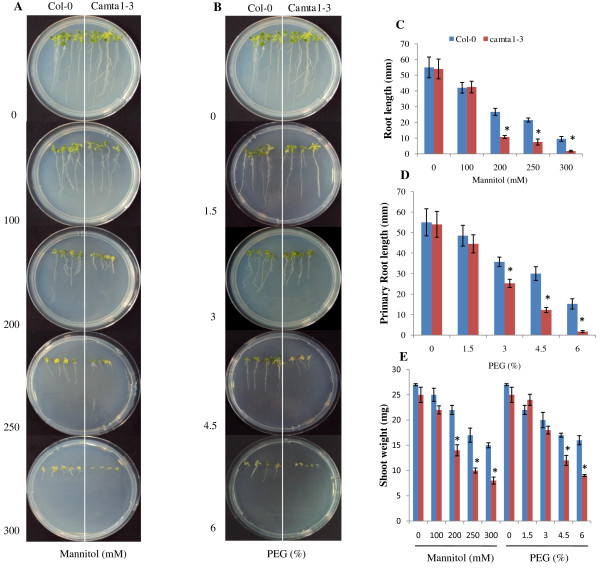
**The reduced drought tolerance of *camta1.*** The one week old seedlings of Col-0 and *camta1-3* were transferred to MS medium (control), (**A**) MS supplemented with mannitol, in mM (100, 200, 250 and 300) and (**B**) MS supplemented with PEG, in % (1.5, 3.0, 4.5 and 6.0) and images were taken after 15 days. The root lengths of the seedlings were measured in millimetre (mm) for (**C**) mannitol stress and (**D**) PEG (poly ethylene gylcol) stress. (**E**) The shoot weights of the seedlings under mannitol and PEG stress were recorded in milligram (mg). Error bars indicates Standard deviation SD (n = 25) and asterisk indicates significantly different from values of Col-0 at P < 0.05 by student’s *t* test.

CAMTA has been reported to play an important role in abiotic stresses in plants especially cold [21], however the role of CAMTA in drought stress was not understood. Thus, to explore and characterise the possible role of CAMTA family of *Arabidopsis* in drought, we obtained homozygous T-DNA insertion lines of all CAMTAs viz., CAMTA1-6 (background columbia-0) from Arabidopsis Biological Resource Centre (ABRC) (Additional file 1). Initially we screened CAMTA mutants by exposing them to various concentrations of mannitol for osmotic stress and their primary root elongation was observed and compared with the Col-0 seedlings (Additional file 2). In control condition, growth rate of all the *camta* were similar to the Col-0 showing no apparent effect on plants caused by the silencing of the CAMTA gene in the mutant. With the increase in mannitol concentration to 300 mM, apart from *camta1*, no significant difference in root growth was observed through *camta2* to *camta6* when compared to Col-0 (Additional file 2). Further, to distinguish the effect of osmotic stress on *camta1*, seedlings of Col-0 and *camta1-3* were allowed to grow vertically on mannitol and PEG (poly ethylene gylcol) concentration series and their shoot weight (SW) and primary root length (RL) were estimated (Figure 1). At 100 mM mannitol and 1.5% PEG, there was no apparent visible phenotypic difference in Col-0 and *camta1-3* growth and their roots architecture (Figure 1A and 1B). At 250 mM mannitol solution, both Col-0 (SW-17 mg; RL-21.5 mm) and *camta1-3* (SW-10 mg; RL-7.5 mm) had stunted growth, particularly *camta1-3* showed higher growth retardation as a sign of growing under the stressed condition (Figure 1A, 1C and 1E). Subsequently, on increasing mannitol concentration to 300 mM, the growth of *camta1-3* (SW-8 mg; RL-1.75 mm) was most severely affected with stunted primary root growth and shoot weight as compared to Col-0 (SW-15 mg; RL-9.5 mm). Similarly, PEG induced osmotic stress showed similar affect on *camta1-3* growth and root development (Figure 1B). At 4.5% PEG, large difference was observed in root and seedling growth of *camta1-3* (SW-12 mg; RL-12.25 mm) as compared to Col-0 (SW-17 mg; RL-30 mm). The PEG-induced (6%) reduction in growth and primary root length was much pronounced in *camta1-3* (SW-9 mg; RL-1.75 mm) than Col-0 (SW-16 mg RL-15.25 mm) (Figure 1B, 1D and 1E). The statistically significant changes have been marked with an asterisk (*) in respective figure (p < 0.05). Error bars indicates Standard deviation SD (n = 25) and asterisk indicates significantly different from values of Col-0 at P < 0.05 by student’s *t* test.

**Figure 2:**
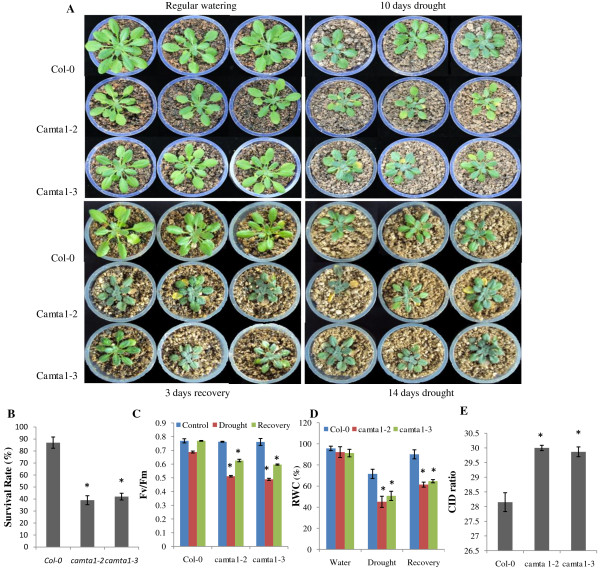
**Performance of *camta1 *plants under drought stress in soil.** Phenotypic variation of the plants under drought stress. (**A**) The Col-0, *camta1-2* and *camta1-3* were grown for 3 weeks under regular water regime followed by withholding water for 14 days, and then re-watered for 3 days. (**B**) Quantitative analysis of the survival rate of the Col-0, *camta1-2* and *camta1-3*. (**C**) The efficiency of photosystem II (Fv/Fm ratio) in the leaves of plants after 14 days of drought stress. (**D**) The relative water content (RWC) in the leaves of plants under drought stress. (**E**) Estimation of water use efficiency (WUE) of Col-0, *camta1-2* and *camta1-3* by carbon isotope discrimination ratio (CID). Error bars indicates Standard deviation SD (n = 20) and asterisk indicates significantly different from values of Col-0 at P < 0.05 by student’s *t* test.

Next, we examined phenotypic variation supported with physiological measurements of 3 week old seedlings of two *camta1* alleles (*camta1-2* and *camta1-3*) and Col-0 in water and drought condition then allowed to recover for 3 days, during which they were watered (Figure 2). Under water condition, Col-0 and both *camta1* mutant (*camta1-2* and *camta1-3*) plants performed equally well. Although both Col-0 and *camta1* mutants showed wilting of leaves under drought condition, the wilting was more pronounced in *camta1-2* and *camta1-3* after 14 days of with-holding water. The *camta1* mutant had stunted round leaves showing wrinkling; appearance of pale yellow and leaves were dried (Figure 2A). The prominent phenotypic variation under drought condition includes significant decrease in rosette leaves count, rosette area, leaf area and primary root length (Additional file 3). After the recovery period of 3 days, camta1 mutant showed poor survivability (39% of *camta1-2* and 42% of *camta1-3*) while more than 86% of Col-0 plants survived and continued to grow after giving water (Figure 2A and 2B). Recently, Water Use Efficiency (WUE) of many plant species were related to isotopic ratio of ^13^C to ^12^C [27]. Thus we determined the isotopic ratio of ^13^C to ^12^C for Col-0 and *camta1* mutant (*camta1-2* and *camta1-3*), the carbon isotope discrimination (Δ) value were 28.15 for Col-0 while 29.99 and 29.86 for *camta1-2* and *camta1-3*, respectively (Figure 2E). The relative increase in (Δ) value of *camta1* mutant than Col-0 showed that it had poor WUE and hence indicates higher sensitivity to drought (P < 0.05). Similarly, Fv/Fm ratio (efficiency of photosystem-II) was also significantly reduced in *camta1-2* (0.511) and *camta1-3* (0.489) than Col-0 (0.687) recorded after with-holding water for 14 days (Figure 2C) (p < 0.05). After rescuing the plant from water-defict condition by rewatering them for 3 days, the increase in Fv/Fm ratio in Col-0 reached at par to the water condition. While *camta1* mutant had poor recovery in Fv/Fm ratio as compared to Col-0. The relative water content (RWC) was reduced by 26% and 20% in *camta1-2* and *camta1-3*, respectively as compared to Col-0 under drought condition (Figure 2D). After rewatering, the RWC of Col-0 reached to 90% while *camta1-2* (61.36%) and *camta1-3* (64.62%) showed decline in RWC. The phenotypic variation, poor WUE, low photosystem II efficiency, decline in RWC and higher sensitivity to drought with reduced survivability of *camta1* can be correlated with the decreased expression level of the CAMTA1 gene in mutant (Additional file 3).The statistically significant changes have been marked with an asterisk (*) in respective figure (p < 0.05). Error bars indicates Standard deviation SD (n = 20) and asterisk indicates significantly different from values of Col-0 at P < 0.05 by student’s *t* test.

### Microarray experimental design to identify CAMTA1 dependent genes

**Figure 3:**
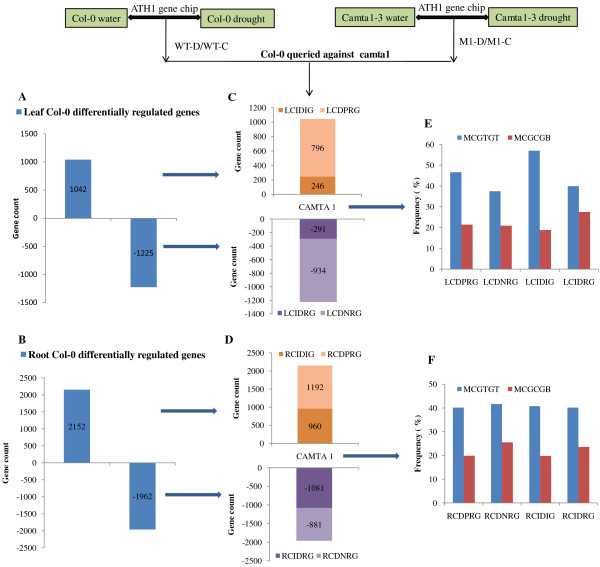
**Pictorial and graphical view of experimental design and analysis.** Total number of differentially regulated genes in Col-0 under drought stress (**A**) In leaf tissue and (**B**) In root tissue. Total number of CAMTA1 regulated genes (**C**) In leaf tissue and (**D**) In root tissue. Distribution of CAMTA recognition motif in frequency (%) across differentially expressed genes (DEGs) (**E**) In leaf tissue and (**F**) In root tissue. The LCDPRG stands for leaf CAMTA1 dependent positively regulated genes, LCDNRG for leaf CAMTA1 dependent negatively regulated genes, LCIDIG for leaf CAMTA1 independent drought induced genes and LCIDRG for leaf CAMTA1 independent drought repressed genes. The RCDPRG stands for root CAMTA1 dependent positively regulated genes, RCDNRG for root CAMTA1 dependent negatively regulated genes, RCIDIG for root CAMTA1 independent drought induced genes and RCIDRG for root CAMTA1 independent drought repressed genes.

**Figure 4:**
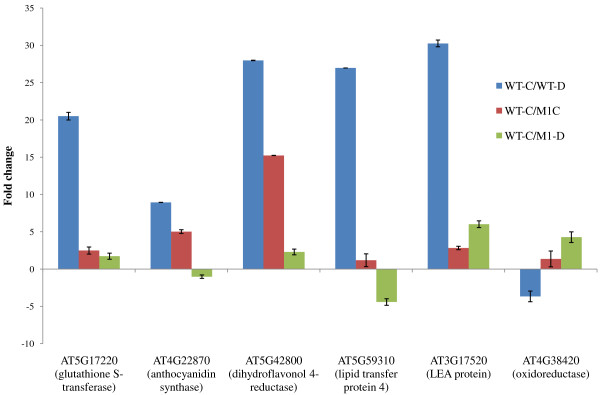
Validation of Microarray differentially expressed genes by RT-PCR.

To understand the global regulation of gene expression by CAMTA1, we used Affymetrix Arabidopsis expression array ATH1 to profile the Col-0 and *camta1-3* expression under drought and water conditions in leaf and root tissue. The ATH1 array contains 22,500 probe sets encoding 24,000 genes/transcripts. Next, by using Significance Analysis of Microarray (SAM), P value ≤ 0.05 and fold change (FC) ≥ 2 were considered as significantly differentially regulated (up or down) transcripts. In Col-0 leaves, 1042 genes were up regulated and 1225 genes were down regulated in response to drought stress (Additional file 4). We identified the status of these genes in *camta1-3* after drought treatment by querying these genes to microarray data of *camta1-3* in leaves (Additional file 5)*.* Out of 1042 genes which were up regulated in Col-0, the expression of 796 genes were significantly down regulated in *camta1-3* (Additional file 6-worksheet 1). These 796 genes were assigned as leaf CAMTA1 dependent positively regulated genes (LCDPRG) (Figure 3A and 3C). The expression of rest of the 246 out of 1042 genes remained unaffected and hence these genes were assigned as leaf CAMTA1 independent drought induced genes (LCIDIG) (Additional file 6-worksheet 3). Further, out of 1225 down regulated genes in Col-0 in response to drought, the expression of 934 genes were significantly changed in *camta1-3* (Additional file 6-worksheet 2) and hence these genes were assigned as leaf CAMTA1 dependent negatively regulated genes (LCDNRG) (Figure 3A and 3C). The expression of remaining 291 out of 1225 genes was not changed significantly and hence these genes were assigned as leaf CAMTA1 independent drought repressed genes (LCIDRG) (Additional file 6-worksheet 4). The 1000 bp upstream promoters sequences of CAMTA dependent/independent genes were then screened for presence or absence of consensus CAMTA1 binding motif (MCGCGB/MCGTGT). The “MCGCGB” *cis*-element was exclusively identified as binding site of CAMTA while “MCGTGT” *cis*-element along with CAMTA was also linked to abscisic acid (ABA) signalling, the ABRE. The LCDPRG had 21.4% genes containing CGCG core motif and 46.6% genes with CGTG core motif. The LCDNRG had 20.9% and 37.5% genes containing CGCG and CGTG, respectively (Figure 3E). The higher number of genes with CGTG core motif could be ascribed to its overlap binding site to ABRE. It was noteworthy that mutation in CAMTA1 resulted in almost 72-75% change in expression of transcriptome controlled by drought. The results indicate that CAMTA1 is one of the global regulators of drought. Similarly, in Col-0 roots under drought condition, 2152 genes were up regulated and 1962 genes were down regulated (Additional file 7). To identify CAMTA1 dependent genes, differentially expressed genes of Col-0 were mapped and queried against *camta1-3* genes (Additional file 8). Out of 2152 differentially up regulated genes in Col-0, the expression of 1192 genes were significantly down regulated *in camta1-3* (Additional file 9-worksheet 1). These 1192 genes were termed as root CAMTA1 dependent positively regulated genes (RCDPRG) (Figure 3B and 3D). The expression of 960 genes out of 2152 genes remained unaffected and hence these genes were assigned as root CAMTA1 independent drought induced genes (RCIDIG) (Additional file 9-worksheet 3). Next, out of 1962 down regulated genes in Col-0 in response to drought, the expression of 881 genes were significantly changed in *camta1-3* (Additional file 9-worksheet 2) and hence these genes were assigned as root CAMTA1 dependentnegatively regulated genes (RCDNRG) (Figure 3B and 4D) while expression of remaining 1081 genes was not much altered hence termed as root CAMTA1 independent drought repressed genes (RCIDRG) (Additional file 9-worksheet 4). The CAMTA recognition *cis*-elements identified revealed higher occurrence of MCGTGT than MCGCGB motif. In RCDPRG 19.9% genes contained MCGCGB while 40.2% genes contained MCGTGT motif and in RCDNRG 25.5% and 41.7% genes had MCGCGB and MCGTGT motif, respectively (Figure 3F). As we were interested in elucidating the role of CAMTA1 protein under drought stress, our further analysis was focussed on CAMTA1 dependent genes. Therefore, in following work and discussion we will be considering LCDPRG, LCDNRG and RCDPRG, RCDNRG.

The expression profiles (fold change value) of genes obtained through microarray were experimentally validated through RT-PCR using 6 genes belonging to dihydroflavonoid 4-reductase, LEA, oxidoreductase, lipid transfer protein, glutathionin s-transferase and anthocyanidin synthase. The results obtained from all the 6 genes tested by RT-PCR agree with the trend of regulation identified by microarray analysis (Figure 4). Thus results of RT-PCR validate the microarray data.

The strategy depicted in Figure 3 (analysis scheme) is to understand the role of CAMTA1 in drought condition which is the unique part of our study. CAMTA1 may have a role in normal physiology, also revealed by the fact that CAMTA1 gene expresses ubiquitously at control and drought condition in *Arabidopsis* (Additional file 10). We made the comparison between WT-C and M1-C and identified that *camta1* does regulate a group of genes since in leaf tissue, 169 genes were down-regulated and 209 genes were up-regulated in *camta1-3* as compared to Col-0 under water condition. In root tissue, 670 genes were up regulated and 635 genes were down regulated. The AgriGO analysis of these differentially expressed genes indicate that the *camta1* under control condition regulates pathways over represented by response to stimulus, response to chemical stimulus, response to organic stimulus substance, response to chitin, response to endogenous stimulus, response to hormone stimulus etc., (Additional file 10). The pathways involved in various regulatory mechanisms were also altered in *camta1-3* under control like transcription regulator activity, regulation of macromolecule biosynthetic process, regulation of primary metabolic process, regulation of nitrogen compound metabolic process etc. (Additional file 10). Alternatively this has facilitated in inferring the tissue specific pathways regulated by *camta1-3* under water condition. The most significant pathway exclusively present in leaf tissue includes transcription regulator activity, response to carbohydrate stimulus, response to chitin etc. The pathways over represented in only root tissue were oxidoreductase activity, regulation of biosynthetic process, metal ion binding etc. (Additional file 10).

### The analysis of CAMTA1 dependent genes for identifying biological processes and pathways regulated by CAMTA1

To elucidate the mechanisms underlying the enhanced sensitivity of the *camta1-3* towards limited water condition, we identified and analysed biological pathways, gene regulation networks and protein interaction maps with CAMTA1 dependent genes by Pathway Studio 9.0 and agriGO (GO Analysis Toolkit and Database for Agricultural Community) analysis tools keeping a stringent cut-off of p-value ≤ 0.05 for identifying significant biological identities. The analysis was carried out firstly with LCDPRG, LCDNRG and RCDPRG, RCDNGR and simultaneously the genes containing CAMTA1-recognition motif were analysed to identify specific *cis*-elements governed potential changes in cellular functions and associated transcriptome interaction networks. This data analysis strategy, in a global and unbiased manner, identifies cellular changes driven specifically by CAMTA1 along with its recognition motif (CGCG or CGTG).

### CAMTA1 dependent positive regulation: Involved in stress response and maintained osmotic balance of cell under drought stress and targets plasma membrane

**Figure 5:**
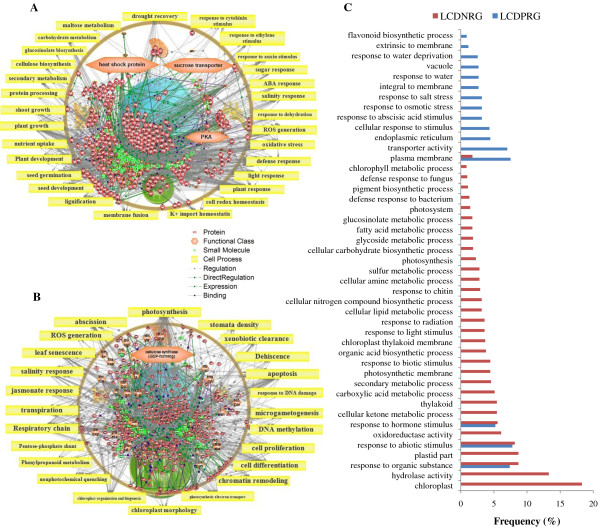
**Pathway analysis of CAMTA1 dependent genes. **(**A**) Leaf CAMTA1 dependent positively regulated genes (LCDPRG). (**B**) Leaf CAMTA1 dependent negatively regulated genes (LCDNRG). (**C**) GO annotation of CAMTA1 dependent genes of leaf tissue (LCDPRG and LCDNRG).

**Figure 6:**
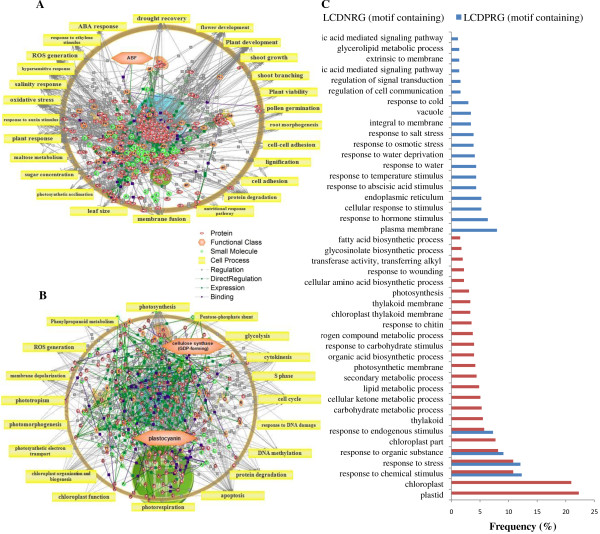
**Pathway analysis of CAMTA1 regulated genes (containing recognition motif). **(**A**) Leaf CAMTA1 dependent positively regulated genes (LCDPRG). (**B**) Leaf CAMTA1 dependent negatively regulated genes (LCDNRG). (**C**) GO annotation of CAMTA 1 dependent genes of leaf tissue (LCDPRG and LCDNRG) containing recognition motif.

**Figure 7:**
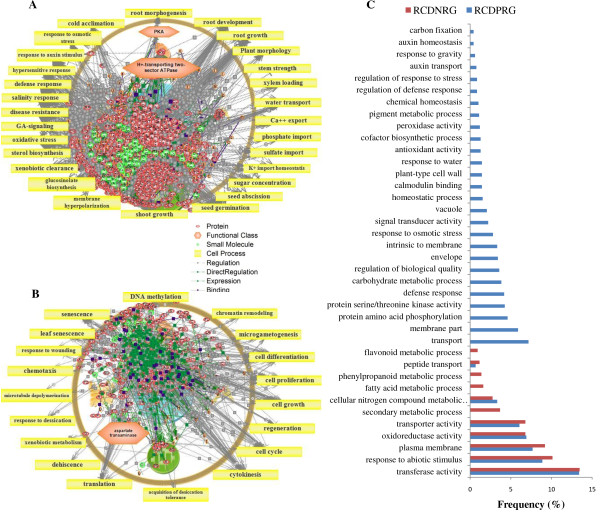
**Pathway analysis of CAMTA1 dependent genes. **(**A**) Root CAMTA1 dependent positively regulated genes (RCDPRG). (**B**) Root CAMTA1 dependent negatively regulated genes (RCDNRG). (**C**) GO annotation of CAMTA 1 dependent genes of root tissue (RCDPRG and RCDNRG).

**Figure 8:**
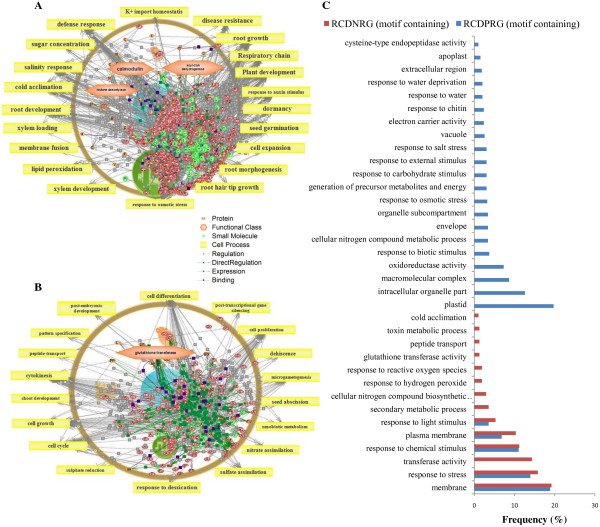
**Pathway analysis of CAMTA1 dependent genes (containing recognition motif). **(**A**) Root CAMTA1 dependent positively regulated genes (RCDPRG). (**B**) Root CAMTA1 dependent negatively regulated genes (RCDNRG). (**C**) GO annotation of CAMTA 1 dependent genes of root tissue (RCDPRG and RCDNRG) containing recognition motif.

The possible functional categories that govern the responses of CAMTA1 dependent positively regulated genes of leaf and root tissue featured several regulators and associate pathways. The most important cell process in LCDPRG includes ‘drought recovery’ (Figure 5A and 6A) and in RCDPRG includes ‘K + import/homeostasis’ (Figure 7A and 8A). The stress adaptation regulated by CAMTA1 for root and leaf tissue influences the pathways related to response to auxin stimulus, hypersensitive response, defense response, plant response, cold acclimation, response to ethylene stimulus, ABA response, salinity response, response to osmotic stress etc. (Figure 5A, 5C, 7A and 7C). CAMTA1 protein probably favored plant growth and development rather than directing it towards cell death and senescence. Various such pathways include root development, plant development, plant morphology, seed germination, flower development, xylem loading, root growth, shoot growth and stem strength (Figure 5A and 7A). The motif-specific (CGCG/CGTG) driven processes for LCDPRG include sugar concentration, plant viability, leaf size, shoot branching, cell adhesion etc. (Figure 6A). For RCDPRG, a motif-specific process includes plant development cell expansion, lipid peroxidation, membrane fusion, root hair tip growth etc. (Figure 8A). The functional class in LCDPRG was Protein kinase A (PKA), heat shock protein, ABA binding factor (ABF) and sucrose transporters while in RCDPRG were H + −transporting two-sector ATPase, calmodulin, histone deacetylase and PKA (Figure 5A, 6A, 7A and 8A). In agriGO, majority of genes were associated to membrane like plasma membrane and membrane part, followed by transporter activity in LCDPRG and RCDPRG (Figure 5A, 5C and 7A, 7C). Other important GO terms modulated in LCDPRG were response to water deprivation, response to osmotic stress, extrinsic to membrane, flavanoid biosynthetic process, integral to membrane etc. (Figure 5C and 6C). The significant GO terms in RCDPRG includes transport, protein amino-acid phosphorylation, response to carbohydrate stimulus, homeostatic process, auxin homeostatic, response to water depriviation, peroxidase activity, signal transducer activity etc. (Figure 7C and 8C).

### CAMTA1 dependent negative regulation: involved in cell differentiation – apoptosis, affect photosynthesis efficiency and targets chloroplast

CAMTA1 dependent negatively regulated genes had several pathways related to cell differentiation and propagation imparting controlled cell division which could be ascribed to decreased rate of cell death (apoptosis) and senescence. Various such cell processes were cell differentiation, DNA methylation, apoptosis, abscission, dehiscence, cytokinesis, cell proliferation, S phase, chromatin remodeling, chloroplast organization and biogenesis (Figure 5B, 7B). The over-represented pathways in LCDNRG were related to leaf anatomy and photosynthesis like, leaf shape, stomatal density, leaf senescence, transpiration, photosynthetic electron transport (Figure 5B). Other important cell processes negatively regulated by CAMTA1 were ROS generation, xenobiotic clearance, fatty acid metabolism, pentose phosphate shunt, microgametogenesis (Figure 5B and 7B). The motif specific cell processes in LCDNRG were chloroplast function, glycolysis, photorespiration, phenyl propanoid metabolism, RNA splicing etc. (Figure 6B). Likewise in RCDNRG, motif specific cell process were post transcriptional gene silencing (PTGS), sulphate assimilates, nitrogen assimilates, seed abscission etc. (Figure 8B). The functional class for LCDNRG includes cellulose synthase, plastocyanin and for RCDNRG includes aspartate transaminase, glutathione transferase (GST) (Figure 5B, 6B, 7B and 8B). The GO analysis of LCDNRG revealed large number of genes related to photosynthesis machinery like chloroplast, plastid, thylakoid, photosystem, photosynthetic membrane etc. (Figure 5C and 6C). The significant GO terms in RCDNRG were response to hydrogen peroxide, glutathione transferase activity, peptide transport, phenylpropanoid metabolic process, fatty acid metabolic process etc. (Figure 7C and 8C).

### CAMTA1 involvement in abiotic stress management

**Figure 9:**
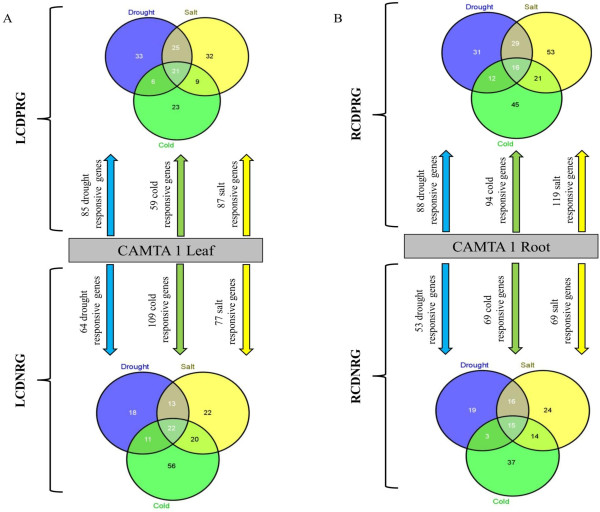
**Different number of abiotic stress responsive genes regulated by CAMTA 1 dependent genes (A) In leaf tissue (B) In root tissue.** The LCDPRG stands for leaf CAMTA1 dependent positively regulated genes, LCDNRG for leaf CAMTA1 dependent negatively regulated genes, RCDPRG stands for root CAMTA1 dependent positively regulated genes and RCDNRG for root CAMTA1 dependent negatively regulated genes.

To monitor the role in various abiotic stress conditions, CAMTA1 dependent genes were scanned to the Stress Responsive Transcription Factor Database (STIFDB) for comprehensive collection of abiotic stress responsive genes. Secondly, to follow the intricate and complicated networks of stress responsive transcription factors activated by CAMTA1 which could be involved in the regulation of these stress responsive genes. In LCDPRG, 85 genes (71.7% genes with CGTG; 21.1% with CGCG), 59 genes (66% with CGTG; 33% with CGCG) and 87 genes (62% with CGTG; 35% with CGCG) were responsive to drought, cold and salt, respectively hence these genes showed positive correlation (up-regulated) in gene expression (Figure 9, Additional file 11-worksheet 3). Whereas in LCDNRG, 64 genes (90.2% genes with CGTG; 20.3% with CGCG), 109 genes (44.1% genes with CGTG; 18.1% with CGCG) and 77 genes (37.6% genes with CGTG; 22% with CGCG) were responsive to drought, cold and salt, respectively and showed negative correlation (down-regulated) in gene expression (Figure 9, Additional file 11-worksheet 3). For RCDPRG, 88, 94 and 119 genes were positively regulated under drought, cold and salt, respectively (Figure 9, Additional file 12-worksheet 3). While for RCDNRG, 53 genes were drought responsive and 59 genes were related to each cold and salinity (Figure 9, Additional file 12-worksheet 3). The occurrence of CGTG consensus sequence was more abundant in 1 kb upstream region due to its binding site to CAMTA1 as well as ABRE. The results indicate the role of CAMTA1 in ABA dependent abiotic stress tolerance.

### CAMTA1 regulate prominent stress responsive genes

We next examined the substantial relationship between the expressions of stress induced transcripts between different abiotic stress conditions. For LCDPRG, 21 stress-responsive genes that responded to all three stress condition had more than 90% similarity with CAMTA1 recognition motif (Figure 9A). Among these we found 5 well established stress inducible genes including early-responsive to dehydration 7 (ERD7) (3-CGCG; 1-CGTG), responsive to ABA 18 (RAB18) (2-CGCG; 5-CGTG), responsive to desiccation 26/22 (RD26/22) (1-CGCG; 6-CGTG), cold regulated 78 (COR78) (1-CGCG; 2-CGTG), low temperature-induced 30 (LTI30) (1-CGTG) (Additional file 11-worksheet 1). These stress induced genes were probably expressed under the influence of CAMTA as they were enriched with CAMTA1 binding *cis*-elements hence presumed to be its direct binding targets. Other important genes showing positive regulation in all 3 stresses were late embryogenesis abundant protein (LEA), cytochrome P450, glyoxylate aminotransferase 3, phosphatase 2CA, ABA-responsive protein, etc. (Additional file 11-worksheet 1). There were 6 genes positively correlated by both drought and cold, 9 genes for cold and salinity and 25 genes regulated by both drought and high salinity. Some genes were unique to their stress condition, 33 genes were exclusively regulated by drought, 23 genes by cold and 32 genes induced only under high salinity (Figure 9A). In LCDPRG, genes modulated only under the drought stress includes NAC TF (1-CGTG; 1-CGCG), MYB TF (5-CGTG), lipid transfer protein 4/3 (LTP4/3) (2-CGTG), glucose phosphate translocator 2 (GPT2) (1-CGTG), UDP-glucosyl transferase 85A5 (2-CGTG; 1-CGCG), scarecrow-like 13 (SCL13), cytochrome P450, AAA-type ATPase, senescence-related gene 1, etc. (Additional file 11-worksheet 1). Expression of genes altered exclusively by salt were WD-40 repeat family protein (WD-40) (1-CGTG; 2-CGCG), GCR2-like 1 (GCL1) (5-CGTG; 2-CGCG), hexokinase 2, jasmonic acid responsive 2, H(+)-ATPase 3, cystatin, etc. (Additional file 11-worksheet 1). Exclusively, cold related genes include expansin A9 (2-CGCG), phosphatidyl serine decarboxylase 3 (1-CGCG), RAP2.2 and 2.1 (2-CGTG), ethylene response sensor 1 (ERS1) (2-CGTG), AP2-TF, copper chaperone, glucose-6-phosphate dehydrogenase 5, etc. (Additional file 11-worksheet 1). Similarly, for RCDPRG, analysis enabled in identifying gene expression with overlapped and/or for specific stress condition. We found 16 genes whose expression was positively regulated under drought, cold and salinity and among these some of the known stress inducible genes driven by CAMTA1 were RD28, nodulin protein, CBL-interacting protein kinase 3, dark inducible 6 (3-CGTG), ferretin 1 (2-CGTG;1-CGCG) (Figure 9B, Additional file 12-worksheet 1). In RCDPRG, 29 genes got affected under both drought and salt, 12 genes by drought and cold and 21 genes got affected by salinity and cold (Figure 9B). Some of the important genes showing positive correlation in drought and salt includes phytoene desaturation 1, RD19 (2-CGTG), acyl-COA oxidase 2 (4-CGTG; 2-CGCG), phytoene desaturation 1 (PDS1) (3-CGTG), MYC2 (1-CGTG; 1-CGCG), tetraspanin 3 (2-CGTG), cinnamyl-alcohol dehydrogenase (1-CGTG), ORA47 transcription factor (1-CGTG; 2-CGCG) (Additional file 12-worksheet 1). Genes that showed altered expression under both drought and cold were ethylene-responsive element binding factor 13 (ERF13) (1-CGCG), C-repeat/DRE binding factor 2 (CBF2) (1-CGCG), dormancy-associated protein 1 (DRM1) (1-CGTG), ABA1 (2-CGTG), WRKY33 (1-CGTG), sensitive to freezing 2, etc. Genes affected under salt and cold includes acetolactate synthase (1-CGTG; 3-CGCG), plasma membrane intrinsic protein 1A, peroxidase 27, etc. (Additional file 12-worksheet 1). There were 31 genes positively modulated exclusively by drought stress like lipoxygenase (1-CGCT; 1-CGCG), protein kinase 19 (3-CGTG; 1-CGCG), glycosyl hydrolase (1-CGTG; 2-CGCG), carboxyesterase 12 (2-CGTG; 1-CGCG), etc. There were 53 and 45 genes specifically related to salt and cold, respectively with positive correlation (upregulated) (Figure 9B)

The LCDNRG had 22 genes negatively correlated (down-regulated) to all 3 stresses (drought, cold and high salinity) and among them majority of genes has been well characterized for stress adaptation like salt tolerance zinc finger (3-CGTG), Arabidopsis NAC domain containing protein 102 (ANAC102) (5-CGTG; 1-CGCG), germin-like protein 1 (1-CGTG), ERD9 (2-CGTG), photosystem I subunit H (PSI-H), carbonic anhydrase1 (Figure 9A, Additional file 11-worksheet 2). The negatively regulated genes had functional redundancy by showing altered expression in more than one stress condition. There were 13 genes modulated under drought and salt condition, 11 genes affected by both cold and drought condition while 20 genes got affected by salinity and cold (Figure 9A). Genes specifically modulated by drought has 18 genes, among them some of the important genes include cinnamoyl CoA reductase 1, auxin-responsive protein, fructose-bisphosphatealdolase, oxidoreductase, tonoplast intrinsic protein 2 etc. There were 22 cold specific genes like Beta galactosidase 1 (BGAL1), MYB91, sugar transporter 1, BTB domain protein 2 (BT2), beta-amylase 9, UDP-glucosyl transferase 73B1 etc. (Additional file 11-worksheet 2). There were 56 genes exclusively affected under cold condition, some of the important genes were MYB77, ERF5, hydrolase 9, Glutathione S-transferase (GST20), ANAC059, ACC synthase 6, etc. (Additional file 11-worksheet 2). In RCDNRG, 19 genes were specifically negatively altered by drought like oxidoreductase, alanine aminotransferase (1-CGCG), DNAJ heat shock protein 20 (HSP20) (1-CGCG), 2-alkenal reductase (1-CGTG; 1-CGCG), beta-ketoacyl-CoA synthase (1-CGTG; 1-CGCG), carboxyesterase 16 (1-CGTG; 1-CGCG), etc. Genes specifically modulated (negative correlation) by cold and salinity were 37 and 24, respectively (Figure 9A, Additional file 12-worksheet 2). In RCDNRG, 15 genes were affected by all the 3 stress condition and majority of them were known stress responsive genes like HSP70, RAB18 (5-CGTG; 2-CGCG), LEA, COR15A, ERD9/10, LTI30, COR47, etc. For either of the 2 stress condition CAMTA1 had 16, 3 and 14 genes affected by drought-salt, drought-cold and salt-cold, respectively (Figure 9B, Additional file 12-worksheet 1).

### Interaction of CAMTA1 with different hormonal pathways

**Figure 10:**
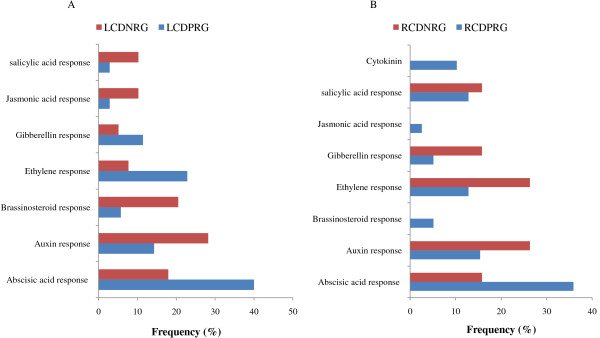
**Expression of different phytohormones associated genes regulated by CAMTA1 (A) In leaf tissue (B) In root tissue.** The LCDPRG stands for leaf CAMTA1 dependent positively regulated genes, LCDNRG for leaf CAMTA1 dependent negatively regulated genes, RCDPRG stands for root CAMTA1 dependent positively regulated genes and RCDNRG for root CAMTA1 dependent negatively regulated genes.

We next examined probable involvement of different phytohormone in co-regulating genes with CAMTA1. Thus, genes involved in biosynthesis and signaling of phytohormones were classified into 8 classes viz, Abscisic, Auxin, Brassinosteroid, Ethylene, Gibberellin, Jasmonic, salicylic, cytokinin identified by Arabidopsis hormone database 2.0 (AHD2). In LCDPRG 35 genes were identified showing positive correlation with various phytohormone associated genes. The response to ABA was most pronounced fetching 40% (14 genes), some of them are known to play role in stress adaptation especially drought like RAB18, heat shock transcription factor C1, WRKY2, phospholipase D, COR78, etc. (Figure 10A, Additional file 13-worksheet 1). The second most affected phytohormone was ethylene contributing 22.8% (8 genes) like ethylene response sensor 1, RD29B, serine-rich splicing factor 31, followed by auxin (14.2%) and gibberlin (11.4%) (Figure 10A). From LCDNRG, 39 genes showed negative correlation to phytohormone associated genes and among them maximum number of genes were associated to auxin constituting 28.2% (11 genes) like MYB77, BT2, auxin-responsive protein 1 (AUX1) (Figure 10A, Additional file 13-worksheet 2). Next, brassinosteroid (BR) was the second phytohormone contributing 8 genes (20.5%) such as BR enhanced expression 3 and 1, Expansin A5, BES1-interacting Myc protein (Figure 10A, Additional file 13-worksheet 2). In RCDPRG, 39 genes were related to phytohormones. Concurrent with the phytohormone analysis of leaf tissue, RCDPRG contain maximum number of genes (35.8%) related to ABA response (Figure 10B). We can postulate that CAMTA1 activated the ABA signalling under drought condition in both leaf and root tissue and hence probably major expression of ABA responsive genes were controlled by CAMTA1 protein. Next, auxin response (15.3%) was generated by CAMTA1 followed by ethylene (12.8%) and salicylic acid (12.8%). Some of the relevant genes associated with ABA includes ABA1 (2-CGTG), ethylene insensitive 2, MYC2 (1-CGTG; 1-CGCG), CBF2 (1-CGCG), rho-related protein 10, etc. Auxin responsive genes co-regulated with CAMTA1 were indole-3-butyric acid response 5, dormancy-associated protein 1, auxin efflux protein, auxin-responsive protein (Figure 10B, Additional file 14-worksheet 1, 2).

### CAMTA1 regulate expression of various global transcription factors involved in abiotic stress

**Figure 11:**
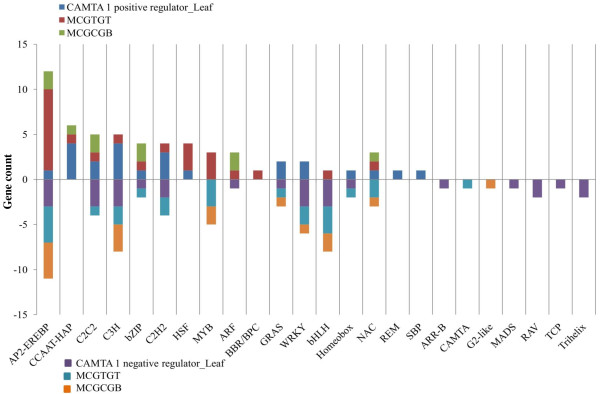
Differentially expressed Transcription Factors regulated by CAMTA 1 in leaf tissue.

**Figure 12:**
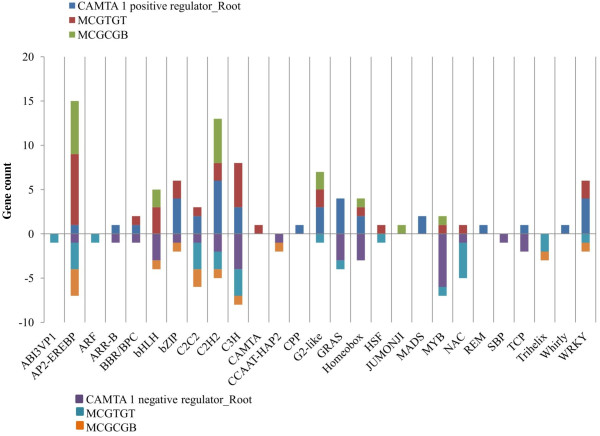
Differentially expressed Transcription Factors regulated by CAMTA 1 in root tissue.

To understand the role of CAMTA1 protein in transcriptional regulation in drought stress, CAMTA1 dependent genes encodes various transcription factors sorted out by Arabidopsis gene regulatory information server (AGRIS). There are known 1,770 transcription factor belonging to 50 families, based on the presence of conserved domains. Out of these 50 families, 24 were targeted by CAMTA1 protein. These 24 TFs regulated by CAMTA1 under drought stress were further grouped on the basis of occurrence of CAMTA1-recognition motif in the 1 kb upstream region of the gene. From the analysis, we obtained 23 AP2-EREBP being over-represented TF in CAMTA1 dependent genes of leaf (12 positively and 11 negatively regulated) (Figure 11). The 83% genes (19 out of 23) encoding AP2 have CAMTA1 recognition site in their promoter region. The AP2 associated genes enriched with CAMTA1 binding site in LCDPRG has 12 genes which annotates 3 DREB member (DREB subfamily A-4, A-5 “RAP2.1” and A-5 “RAP2.10”) 7 ERF (2 genes of subfamily B-3, B-6, B-2, and 1 gene of subfamily B-4 member) and other 2 were AP2 domain protein (Additional file 15-worksheet 1). There were 11 genes encoding AP2 in LCDNRG which includes 6 ERF (6 genes of subfamily B-3), 4 DREB member (2 genes of subfamily A-6 “RAP2.4” and gene of subfamily A-1 and A-5) and 2 AP2-domain protein (Additional file 15-worksheet 2). The annotation of various genes of AP2 and presence of CAMTA1 binding site clearly indicates that CAMTA1 target AP2 associated genes in a specific manner by binding to distinct set of subfamily in highly specific manner. MYB was another TF important for drought tolerance. The probable TF acting as positive regulator for CAMTA1 activation could be Heat shock protein factor (HSF) (4 genes; ATHSFA1E, A1B, A6A, C1), squamosa binding protein (SBP) and CCAAT-HAP (6 genes; NF-YA7, 5, 3, 2, 9, 1), as they were exclusively present in positively regulated genes of leaf and contain CAMTA1 binding site in their promoter region (Figure 11, Additional file 15-worksheet 1). While TF acting as negative regulator of CAMTA1 could be BHLH as 8 genes associated to BHLH (AtbHLH44, 50, 154, 47, 57, 58, 46, 137) were present in LCDNRG containing 3 genes with CGTG and 2 with CGCG binding motif (Additional file 15-worksheet 2). Concurrent with leaf tissue, in RCDPRG, AP2-EREBP established maximum connection with the CAMTA1 showing large number of associated genes (Figure 12). There were 15 members of AP2 which were positively regulated (like DREB1C, ATERF-2, 11, 13, 15, Rap2.12 etc.) and 7 members were negatively regulated by CAMTA1 (Additional file 16). The WRKY (WRKY17, 33, 70, 53, 75, 74), G2-like (like SCL3, GRAS2) and C2H2 acted as positive regulator whereas MYB (MYB63, 14, 305, 109, 87, 4) acted as negative regulator (Additional file 16).

### The transcriptome of *camta1-2* and *camta1-3*

The transcriptome of *camta1-2* by Galon et al., 2010 (published data) was compared with the gene expression profiling of *camta1-3* (our data) and has a correlation of r = 0.86 (Additional file 17). The 63 genes were commonly up-regulated while 33 genes were commonly down regulated in both *camta1-2* and *camta1-3* as compared to wild type *Col-0*. Some of the interesting commonly expressed genes have been listed in additional file 17. The expression of chalcone synthase, UDP-glucoronosyl, polygalacturonase, early light-inducable protein, cysteine proteinase, ethylene-responsive element-binding family protein, disease resistance protein etc. were found to be repressed in both *camta1-2* and *camta1-3*. Similarly, some of the commonly up regulated genes include mannitol transporter, wall-associated kinase, glycine-rich protein, cytochrome p450, ARR15 (auxin response regulator15), WRKY26 etc. (Additional file 17). As reported in Galon et. al, 2010 study [19], the transcriptome comparative study between two alleles of *camta1* mutant (*camta1-1* and *camta1-2*) through Mapman showed similar pathways when analysed for *camta1-3*. The similar pathways between *camta1-3* and *camta1-2* includes cytokinin metabolism, metabolism of sulphur containing compounds, flavanoids (Additional file 17). Therefore the transcript profiling of *camta1-3* was in concurrent with earlier reported pathways affected by *camta1* mutant.

## Discussion

This is the first report elucidating the role of CAMTA1 gene in drought stress, exploring through the transcript analysis of the *camta1-3* mutant. We identified that *camta1* was most susceptible to drought stress (Figure 1 and 2). The most striking difference was that *camta1-2* and *camta1-3* showed inward adaxial rolling of leaf and severe loss of chlorophyll showing apparently damaged yellow to purplish-black appearance and stunted growth which revealed enhanced effect of drought on *camta1* as compared to Col-0 (Figure 2A). The different abiotic stress response of plants depends on root growth and the stage of development [28]. Therefore, we tried to determine the effect of osmotic stress on *camta1* by root bending assay. Under osmotic stress (mannitol and PEG), the reduction in shoot weight and inhibition of root growth was more in *camta1-3* as compared to Col-0 (p < 0.05). At 300mM mannitol and 6.5% PEG, significant growth retardation in terms of rosette leaves, shoot weight and primary root length could be ascribed to the silencing of the CAMTA1 gene in mutant (knock-out mutant) (Figure 1). Thus, we hypothesize that CAMTA1 acts as positive regulator of plant growth under drought stress based on our observation in osmotic and drought stress experiment in soil (Figure 1 and 2, Additional file 3). After rewatering, the *camta1* mutant (*camta1-2* and *camta1-3*) exhibited growth inhibition, whereas the Col-0 plants thrived well indicating that loss of functional CAMTA1 protein in mutants had a negative role against drought stress (P < 0.05). The carbon isotopes discrimination (CID), relative water content (RWC) and photosystem II efficiency (Fv/Fm ratio) can prove an important criterion for the selection of plant with variable drought tolerance. According to Farquhar GD et.al, a shift in the CID ratio of plant gives information about the plant water use efficiency (WUE) and indicates the plant inherent trait to adapt under stress condition and confirms the stress induced changes in the ^12^C/^13^C ratio [27]. The variation between the CID values of Col-0 and *camta1* indicates that the plant significantly discriminate between heavier and lighter carbon during photosynthesis (Figure 2E). Earlier report states that the variation in CID was known to arise from variation in photosynthetic capacity, stomatal conductance, WUE [29]. The reduction in Fv/Fm ratio might primarily be due to decline in RWC. The low WUE, RWC and decreased efficiency of photosystem II of the *camta1* attributes to the poor tolerance of plant for the drought stress which may be due the loss of CAMTA1 function in the mutant suggesting its probable role in stress tolerance. In brief, we hypothesized that *camta1* apparently plays a role in the natural plant development under stress condition, because its mutation clearly results in stunted plant growth with altered root development and increased sensitivity to osmotic stress. The data indicates that the CAMTA1 gene is required for stress responses that improve drought tolerance through various response mechanisms (Additional file 10). The disruption of functional CAMTA protein in mutant resulted in alteration of various regulatory pathways and stress responses (Additional file 10). To broaden our knowledge horizon for the role of CAMTA1 gene, the comparative analysis of gene profiling under drought and control condition of Col-0 and *camta1-3* was studied. The transcript analysis showed decreased gene count in *camta1-3* than Col-0 under drought condition (Additional file 4 and 7). This clearly reflects that expression of large number of stress induced transcripts were reduced to non-significant level (FC ≤ 2) due to the disruption of the CAMTA1 protein in the mutant resulting in masked expression of its respective target genes. Secondly, the increase in gene count in Col-0 was due to drought stress imposed on plant which depicts large number of genes have undergone reprogramming under drought stress (Additional file 5, 8).

To identify in equitable and unprejudiced way, genes regulated by CAMTA1, the genes were selected on the basis of their non-significant expression level in *camta1-3* with respect to its respective significant expression in the Col-0. Thus CAMTA1 dependent genes were classified as either positively regulated viz., LCDPRG and RCDPRG or negatively regulated viz., LCDNRG and RCDNRG. Motif-analysis facilitated in identifying CAMTA1 binding site in various abiotic stress, phytohormone and TFs related genes which could be later used in establishing their binding affinity to the CAMTA1 *cis*-element (MCGCGB, MCGTGT) (Figure 3E and 3F). The pathway analysis strategy, in a global and unbiased manner, identifies cellular changes driven by specific CAMTA1 recognition motif genes. The most distinguished cell process was “drought recovery” as it clearly indicates the potential nature of CAMTA1 protein to combat and recover under drought stress (Figure 5A and 6A). Drought stress does not affect the plant in isolation but comes in combitorial with other stress condition. For the plant to sustain drought stress, CAMTA1 protein channelizes several stress responsive cell processes and develops plethora of responses that might help the plant to acclimatize and survive in the stressed environment. The higher number of stress responsive genes, signal sensors and transporters in LCDPRG and RCDPRG indicates the expression of more genes associated with stress mechanism. Signal transduction and transporters play major role under drought condition by maintaining osmotic homeostasis, operates the signalling and growth development pathways (Figure 5A and 7A) [30]. The genes encoding plasma membrane and its constituents acted as positive regulator indicating the presence of several genes associated to membrane integrity and biogenesis which were involved in the formation, organization, maintenance of the membrane and in turn protects the cell against mechanical damage, osmotic strength (Figure 4C and 5C) [28]. The higher expression of these under the influence of CAMTA1 maintains membrane structure and preserve cell compartmentation and by synthesizing constituent macromolecules under drought condition provides rapid tolerance to stress [31]. The accumulation of flavonoid is a trademark of plant stress [32] which aims at countering the generation of ROS and leads to the inactivation of antioxidant enzymes constituting a secondary ROS-scavenging system in plants which are exposed to stress conditions (Figure 5C) [33]. Thus positive regulation of the flavonoid biosynthesis imparts tolerance to the plant when exposed to the drought condition. Photosynthesis plays a pivotal role in plant performance under drought. In LCDNRG, there were several genes related to photosynthesis, stomata, chlorophyll, heme, FAD and transpiration (Figure 5B, 5C and 6B). The decline in photosynthesis machinery results in lower net carbon uptake in leaf under water deficit condition which is followed by an alteration in partitioning of the photoassimilates at the plant level, consequently leads to an increase in the root to shoot ratio (Figure 5B, 5C, 6B and 6C) [34]. This is the prima facie for the maintenance of root growth under decreasing water in the soil. In general, this response is mediated by phytohormone, namely by abscisic acid (ABA) [35]. The CAMTA1 generates response to ABA and auxin which induces lateral root formation for optimal water uptake as profilic root system is vital for drought tolerance (Figure 7A). Tightly regulated expression of phytohormone under drought condition determines lateral root meristem activation via an ABA-auxin signalling crosstalk and ethylene (Figure 10A) [36]. In previous reports growth promotion is considered a specific feature for ethylene response, so to establish equilibrium, plant optimize growth and tolerate stress response which involves the synthesis of ethylene [37]. During drought stress response, ABA regulates stomatal aperture and leads to activation of several genes and secondary messengers, including calcium, Inositol trisphosphate, cADP, ribose, etc. [32,38]. Hence conjugated effect of phytohormones induced development and photosynthetic regulation directs the plant for survival in stress environment**.** The presence of ABA indicates the concerted action of CAMTA1 in cell signalling which progressively leads to a massive reprogramming to combat stress (Figure 10A and 10B). Heat shock proteins (HSPs), known as molecular chaperon, rapidly accumulates under stress condition and play a major role in protein folding (Figure 5A) [39]. Numerous studies on histone and DNA methylation highlights its key role in gene expression and plant development under stress [40]. Apart from its role in development under stress, DNA methylation was also associated with gene silencing and transposon control in plant and fungi [41]. Recent studies indicate that transcriptional gene silencing and post transcriptional gene silencing (PTGS) were mechanistically related because they were correlated to same events, including changes in DNA methylation [42]. The cell differentiation, propogation and reprogramming were governed by major changes in the epigenome [43]**.** CAMTA1 acts as a negative regulator for the DNA methylation, gene silencing, apoptosis, cell proliferation, PTGS. Hence by negatively regulating the epigenetic mediated gene silencing and cell differentiation, CAMTA1 probably decreases the rate of silenced gene and PTGS and allows the expression of several genes potential for generating appropriate cellular responses which could be otherwise masked by the effect of DNA methylation (Figure 5B and 7B). Secondly by inhibiting DNA methylation and cell differentiation, CAMTA1 tightly regulates genetics events governing plants death or else the cell differentiation could eventually leads to developmental cell senescence [44]. Therefore by acting as a negative regulator to DNA methylation and cell proliferation CAMTA1 protects the plant from untimely stress induced senescence and directs the expression of stress responsive genes. Study on cellulose synthase by Chen Z et.al in 2005 revealed that its mutant were more tolerant to drought stress as well as to NaCl, mannitol and found higher accumulation of osmolytes and ABA in mutant than Col-0 [45]. Hence decrease in cellulose synthase gene expression enhances drought tolerance which was in concurrent with the reduction of the cellulose synthase by CAMTA1 (Figure 6B). The LEA proteins protect various macromolecules, such as enzymes and lipids, from dehydration [46].

**Figure 13:**
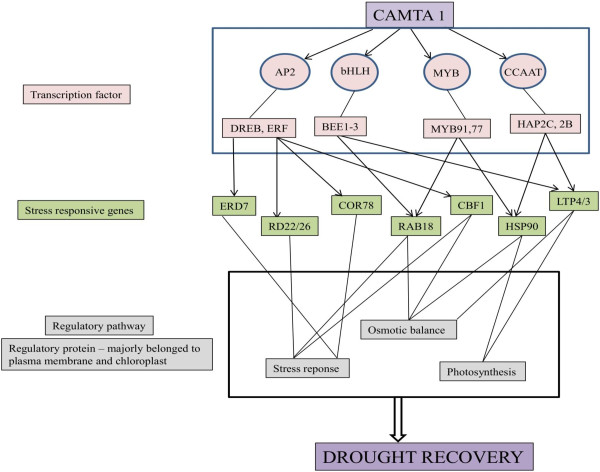
Proposed role of CAMTA1 in the drought stress environment.

The stress responsive genes such as ERD7, RAB18, RD22, COR78 encoded the protein which helps in protecting cells from water deficit and regulating genes for signal transduction and gene expression in the water stress response (Additional file 11, 12) [30,33,47-49]. These genes have been earlier reported as ABA-responsive like DREB, LEA proteins and RAB16 in rice [32], dehydrins in barley [50], and RD29 in *A.thaliana*[51]. These “stress responsive genes” were rapidly induced by stress conditions [32]. Several transcription factors such as DREB1B and DREB1C govern the stress regulations and contain the AP2 domain and bind to the DRE/CRT motif in drought, high salt and cold stress-responsive gene promoters, mediates their downstream gene expression to help the plant survive in a stressed environment (Figure 11 and 12) [49]. Earlier studies revealed that many drought-responsive genes such as RD29A, LTI, COR78, and RD26 and RAB18 were also induced by cold stress in a DREB1A-dependent manner. These genes were probably expressed under the influence of CAMTA1 as they are enriched with CAMTA1 binding *cis*-elements hence presumed to be its direct targets (Additional file 11, 12). Large numbers of drought induced genes were also induced by salinity, which indicates a strong correlation between drought and salt signaling response generated by CAMTA1 dependent genes (Figure 9A and 9B). This depicts CAMTA1 capability to adapt to dual stress condition and indicates the existence of cross-talk between drought and salinity response. It has been documented that biosynthesis and accumulation of ABA, salicylic acid, jasmonic acid and auxin phytohormone are closely linked with drought resistance responses in many plant species. The associated TFs of ABA signaling such as MYB and WRKY cascades the drought stress response and modulates the biosynthesis of various secondary metabolic pathways in response to drought by regulating gene expression (Figure 11). As part of the regulation of drought stress responses, ABA may interact with jasmonic acid and stimulate stomatal closure, while its regulation of gene expression includes the induction of genes associated with response to ethylene, cytokinin, or auxin. Jasmonic and salicylic acid signalling response were enriched in LCDNRG (Figure 10B). The CAMTA1 dependent TFs likely represent key elements in the ability of the CAMTA1 to modify gene expression as part of the plant drought response. The expression of 12 AP2, 4 bZIP, 3 MYB, 2 WRKY, and 2 GRAS TFs genes were positively regulated by CAMTA1 (Figure 11 and 12). Members from these TF families were previously shown to modulate response of plant drought stress [21]. The TFs belonging to these families interact with specific *cis*-elements and/or proteins; and their over expression confers stress tolerance in heterologous systems [28,52]. Among the TFs, ERF subgroups (ERF-B2, B3, B-6) HSF, WRKY and C2H2 were induced and previous reports stated their ability to regulate osmotic and drought responses (Additional file 15, 16) [53]. CAMTA1 positively induced the expression of these transcription factor and others drought responsive genes, probably by binding to CGCG and CGTG boxes in promoter region of these target genes (Figure 10 and 11). Likewise the expressions of the different member of same family responded negatively under drought stress for example, 11 AP2, 10 bZIP, 5 MYB, and 5 WRKY. Overproduction of either AP2 or other regulatory proteins in plants resulted in deleterious effects on plant growth and development [54]. In nature, AP2, bZIP and MYB exhibit stress-inducible expression patterns in response to various stresses such as drought, salt and cold [28]. Therefore the down regulated expression of these transcription factors restricts the expression of various genes and control the levels of regulatory proteins for maintaining the homeostasis of plants. The in-depth interrogation of CAMTA1 dependent genes lead us to hypothesis a biological network involved in drought stress guarded by CAMTA 1 (Figure 13). It indicates that majority of CAMTA1 dependent genes were localized to plasma membrane and chloroplast which positively regulates stress response and osmotic balance while negatively involved in photosynthesis. These regulatory pathways were channeled through various adaptative and stress responsive genes like RAB18, COR78, CBF1, ERD7 etc. The expressions of these genes were controlled by TFs like DREB, bHLH, MYB etc., which in turn were activated by CAMTA1 (Figure 13).

## Conclusion

The results establish a role for CAMTA1 in drought acclimation and provide a possible point of integrating various molecular and biological pathways with drought stress regulated gene expression. The interaction with several stress responsive genes, maintenance of osmoticum, regulating membrane biogenesis, generating ABA response, guarding photosynthesis and interaction with AP2-EREBP were some of the key regulatory components of CAMTA1 in response to drought stress. These findings provide insight for further investigation of CAMTA1 function under drought stress and open new perspectives for improving drought tolerance which could eventually lead to better crop production.

## Methods

### Plant material and treatments

The Col-0 *Arabidopsis thaliana* (Columbia-0 ecotype) and homozygous T-DNA insertion line of *AtCAMTAs* (background Columbia-0) was obtained from the Arabidopsis Biological Resource Centre (ABRC, the Ohio State University, Columbus, OH, USA) (Additional file 1). To evaluate the osmotic sensitivity of Col-0 and *camta1-3*, seeds were surface sterilized in 4% (v/v) sodium hypochlorite for 5 min, washed 8 times with sterilized water. The Murashige and Skoog (half strength; 1/2 MS) medium with 1% (w/v) sucrose, 0.8% (w/v) agar and pH 5.7 was prepared. The seedlings from Col-0 and *camta1-3* were cultured in MS medium for one week and then transferred to MS (control) or MS supplemented with variable concentration of mannitol (100, 200, 250 and 300 mM) and PEG (1.5, 3.0, 4.5 and 6%) solutions and allowed to grow vertically along with control for 14 days. The seedlings growth (shoot weight) and root length was measured and photographed. The experiment was performed in triplicates.

To study the response of Col-0 and *camta1* mutant for drought stress, seeds of Col-0, *camta1-2* and *camta1-3* were placed in solerite soil in pots and incubated for 2 days at 4°C before being transferred in growth chamber for growth at 22°C with illumination at 120 μmol m^-2^ s^-1^ for a 16-h daily light period. The relative humidity was approximate 70% to 65%. The 3 week old seedlings were subjected to drought treatment by withholding water for 14 days (soil moisture below 30%) and control plants were watered every alternate day. After the drought stress of 14 days, plants were watered and allowed to recover for 3 days, and survival rates were then calculated. The carbon isotope discrimination ratio was estimated as described by Farquhar GD et.al [27]. For determination of relative water content (RWC), fresh leaves of Col-0, *camta1-2* and c*amta1-3* were detached and weighed immediately to record FW, followed by dipping them in distilled water for 12 h. The leaves were then blotted, weighed to record turgid weight (TW), and subjected to oven drying at 70°C for 24 h to record dry weight (DW). The RWC was determined by the equation:

$$\mathrm{RWC}=\left(\mathrm{FW-DW}\right)\times 100/\left(\mathrm{TW-DW}\right)$$

The efficiency of photosystem II (Fv/Fm ratio) was determined on first or second leaves from the tip of branches, using MAXI-version of Imaging-PAM (Walz, Effeltrich, Germany).

### Statistical analysis

All experiments were performed at least three times independently. Results were assessed by Student’s t test. Significance was defined as P < 0.05. The statistically significant changes have been marked with an asterisk (*) in respective figure (p < 0.05).

### RNA extraction, cRNA preparation, microarray hybridization and processing

A total 24 samples were processed for Affymetrix gene chip ATH1 analysis. The ecotype Col-0 and *camta1* mutant were grown under drought stress and water condition. Three biological replicates sample were collected from *camta1-3* and Col-0 in watered and drought stressed condition (when drooping effect in leaves were observed and soil moisture was below 30%). Leaf tissues were directly harvested for RNA isolation at 10th day of drought stress. Plants were up-rooted and roots were grinded in liquid nitrogen for RNA isolation. Total RNA was extracted from leaf and root tissue of Col-0 and *camta1-3* using the Spectrum Plant Total RNA Isolation kit (Sigma-Aldrich, USA) by incorporating a DNaseI treatment step using RNase-free DNaseI set (Ambion), according to the manufacturer’s instructions. Total 24 samples were prepared which comprises 3 leaf samples of each Col-0 and *camta1-3* in water condition and drought condition (3x2x2 = 12), similarly 3 root samples of Col-0 and *camta1-3* in water and drought condition (3x2x2 = 12). A total of 250 ng of each RNA was subjected to cDNA/double strand DNA synthesis using one-cycle cDNA Synthesis Kit (Affymetrix, Inc., Santa Clara, CA). The biotin-labelled nucleotides were incorporated during the second step in vitro transcription reaction by using the gene chip IVT Labelling Kit (Affymetrix). The resulting labelled anti-sense RNA samples were fragmented and 15 μg each per array was hybridized to 24 gene chip, ATH1 Arabidopsis Genome Arrays (Affymetrix) for 16 h at 45°C. Once completed, arrays were processed according to the manufacturer’s protocol and scanned using the Gene Chip® Scanner 3000 (Affymetrix).

### Microarray data analysis, identification of CAMTA dependent genes and its analysis

The Arabidopsis gene chip ATH1 Genome array (Affymetrix) contains more than 22,500 probe sets corresponding to approximately 24,000 transcripts. The arrays images were first quantified using Gene Chip Operating Software (GCOS, Affymetrix). The Affymetrix Arabidopsis genome array *cel* files were analysed by Array Assist Software 5.2.2 (Agilent Technologies, Santa Clara, CA, USA). The Affymetrix microarray data is submitted to GEO with accession series GSE40061. The GC-RMA algorithm with quantile normalization was used to summarize the probes from the arrays [55]. Differentially expressed genes with a detection p-value less than 0.05 (p-value ≤ 0.05) and fold change greater or equal to 2 (FC ≥ 2) were considered significant in three biological replicate experiments. The GraphPad prism5 (http://www.graphpad.com/prism) software was used for the identification of CAMTA1 dependent genes, for this, ratio between the fold change of differentially regulated gene list of *camta1-3* (M1-D/M1-C) and Col-0 (WT-D/WT-C) were taken as input for this software. The column statistics analysis was performed which computes descriptive statistics (and normality tests) for each gene. The ratios of genes with values greater or equal to threshold value (99% of confidence of interval) were defined as CAMTA1 dependent genes. The *de-novo* computational identification of CAMTA1 binding sites (MCGTGT and MCGCGB) in a set of upstream regions (1000bp) of CAMTA1 regulated genes was performed by Suite for Computational identification Of Promoter Elements (SCOPE) [56]. CAMTA1 positively and negatively regulated gene list were taken as input in SCOPE for searching particular binding sites related genes. Pathway analysis of CAMTA1 positively and negatively regulated genes was done with Pathway Studio software 9.0 (http://www.ariadnegenomics.com). The TAIR ID of these genes was taken as input for shortest path algorithm in pathway studio. Proteins, metabolites (or small molecules), functional classes and cell processes were taken as entity type for establishment of pathway by using plant ResNet 4.0 database (Ariadne Genomics) employing four interaction type namely regulation, direct regulation, expression and binding [57]. Genes without interactions with others were removed according to the original references recorded by the software. The Gene Ontology (GO) analysis was done with AgriGO (bioinfo.cau.edu.cn/agriGO/index.php) for CAMTA1 dependent genes of leaf and root tissues. The numbers of significant GO terms were large; therefore multiple testing method was performed in order to control the rate of errors. We used SEA (Singular Enrichment Analysis) algorithm, which performs the Fisher statistical test method and by default Benjamini–Yekutieli method with 0.05 significance level to do the multiple comparison correction is used. The Abiotic stress related data was obtained from STIFDB (Stress Responsive Transcription Factor Database) (http://caps.ncbs.res.in/stifdb) and genes related to drought, salt and cold were retrieved [58]. CAMTA positively and negatively regulated genes were queried to the gene list with stress related data. Arabidopsis hormone related genes were downloaded from Arabidopsis Hormone Database 2.0 [59]. TAIR ID of CAMTA1 positively and negatively regulated genes from leaf and root tissues were taken as input for searching particular hormone related gene. On the similarity search basis genes were grouped them into respective phytohormone. Retrieval of particular transcription factor related genes was done by AGRIS [60]. Frequency of CAMTA1 positively and negatively regulated genes was calculated on similarity basis with the locus IDs of these genes to the IDs of transcription factor related genes present in this database.

### Validation of microarray data using RT-PCR

Following total RNA extraction from all 24 samples, cDNA was synthesized in a 40 μl reaction volume using SuperScript ® III reverse transcriptase kit (Invitrogen) supplemented with 200 ng of random primers (Invitrogen) according to the manufacturer’s instructions. The cDNA synthesis reaction conditions were 70°C for 5 min, 25°C for 5 min, 50°C for 1 h, followed by heat inactivation of the enzyme at 75°C for 15 min. Relative transcript abundance of selected genes were assessed by performing RT-PCR using the ABI Prism 7300 Sequence Detection System (Applied Biosystems Foster City, CA, USA). ). Primer sequences used in reactions are described in Additional file 18 and reaction of RT-PCR was performed in a 10 μl reaction volume by adding 0.5 μl cDNA aliquot of each sample to the PCR mix containing gene specific primers and 50% SYBR® Green PCR Master mix. Quantification of transcript (mRNA expression) levels was carried out by using the ΔΔCt quantitative methods. Normalization was carried out by subtracting the ΔΔCt values of ubiquitin from the corresponding ΔΔCt values of the target gene. Following normalization the relative abundance of transcript was calculated from the expression ratios to calculate a fold change value.

## Abbreviations

CAMTA: Calmodulin binding transcription activator; LCDPRG: Leaf CAMTA1 dependent positively regulated genes; LCDNRG: Leaf CAMTA1 dependent negatively regulated genes; LCIDIG: Leaf CAMTA1 independent drought induced genes; LCIDRG: Leaf CAMTA1 independent drought repressed genes; RCDPRG: Root CAMTA1 dependent positively regulated genes; RCDNRG: Root CAMTA1 dependent negatively regulated genes; RCIDIG: Root CAMTA1 independent drought induced genes; RCIDRG: Root CAMTA1 independent drought repressed genes.

## Competing interest

All authors read and approved the manuscript and declare no competing interest.

## Authors’ contribution

NP carried out the stress treatment experiments for screening of plant, sample preparation, performed microarray experiment, RT-PCR, microarray data analysis and drafted the manuscript. AR helped in microarray, initial microarray data analysis and data interpretation. PP helped in data analysis. RKT helped in RT-PCR. FA helped in initial stress experiment. UVP monitored the physiological data. HPP participated in manuscript revision. SVS mentored the entire project, designing of problem and critically revised the manuscript. All authors read and approved the final manuscript.

## Supplementary Material

Additional file 1 **Detailed information of ATCAMTA1-6 mutant with T-DNA insertion site**. Click here for file

Additional file 2 **Screening of different CAMTA mutant for drought tolerance.** By using different mannitol concentration in MS media by root bending assay to show differential primary root growth of Col-0 and CAMTA mutant. (**A**) Col-0, (**B**) *camta1,* (**C**) *camta2,* (**D**) *camta3,* (**E**) *camta4,* (**F**) *camta5,* (**G**) *camta6*. Click here for file

Additional file 3 **The phenotypic characterisation of *camta1 *mutant and Col-0 under drought stress and the relative expression of CAMTA1 in mutant and Col-0.** Quantitative phenotypic measurements of Col-0, *camta1-2* and *camta1-3.* The 3 weeks old plants were subjected to drought stress and after 14 days of stress the measurements have been recorded. Data are given as averages ± SD for 25 plants.The Expression of CAMTA1 gene in Col-0 and *camta1-2* and *camta1-3* by RT-PCR. Click here for file

Additional file 4 **Gene description of differentially regulated genes in leaf tissue of Col-0.** List of up-regulated genes of leaf tissue in WT-D/WT-C_L (Fold change ≥ 2) (work sheet 1). List of down-regulated genes of leaf tissue in WT-D/WT-C_L (Fold change ≥ 2) (work sheet 2). Click here for file

Additional file 5 **Gene description of differentially regulated genes in leaf tissue of *camta1-3*.** List of up-regulated genes of leaf tissue in M1-D/M1-C_L (Fold change ≥ 2) (work sheet 1). List of down-regulated genes of leaf tissue in M1-D/M1-C_L (Fold change ≥ 2 ) (work sheet 2). Click here for file

Additional file 6 **List of CAMTA1 dependent genes of leaf tissue.** List of leaf CAMTA1 dependent positively regulated genes (LCDPRG) sorted in accordance with its 2 recognition motif (MCGTGT; MCGCGB) (work sheet 1). List of leaf CAMTA1 dependent negatively regulated genes (LCDNRG) sorted in accordance with its 2 recognition motif (MCGTGT; MCGCGB) (work sheet 2). List of leaf CAMTA1 independent drought induced genes (LCIDIG) sorted in accordance with its 2 recognition motif (MCGTGT; MCGCGB) (work sheet 3). List of leaf CAMTA1 independent drought repressed genes (LCIDRG) sorted in accordance with its 2 recognition motif (MCGTGT; MCGCGB) (work sheet 4). Click here for file

Additional file 7 **Gene description of differentially regulated genes in root tissue of Col-0.** List of up regulated genes of root tissue in WT-D/WT--_R (Fold change ≥ 2) (work sheet 1). List of down regulated genes of root tissue in WT-D/WT--_R (Fold change ≥ 2) (work sheet 1). Click here for file

Additional file 8 **Gene description of differentially regulated genes in root tissue of *camta1-3*.** List of up-regulated genes of root tissue in M1-D/M1-C-R (Fold change ≥ 2) (work sheet 1). List of down-regulated genes of root tissue in M1-D/M1-_R (Fold change ≥ 2) (work sheet 2). Click here for file

Additional file 9 **List of CAMTA1 dependent genes of root tissue.** List of root CAMTA1 dependent positively regulated genes (RCDPRG) sorted in accordance with its 2 recognition motif (MCGTGT; MCGCGB) (work sheet 1). List of root CAMTA1 dependent negatively regulated genes (RCDNRG) sorted in accordance with its 2 recognition motif (MCGTGT; MCGCGB) (work sheet 2). List of root CAMTA1 independent drought induced genes (RCIDIG) sorted in accordance with its 2 recognition motif (MCGTGT; MCGCGB) (work sheet 3). List of root CAMTA1 independent drought repressed genes (RCIDRG) sorted in accordance with its 2 recognition motif (MCGTGT; MCGCGB) (work sheet 4). Click here for file

Additional file 10 **The comparison between Col-0 and *camta1-3 *under water condition. **(**A**) The relative expression of CAMTA1 gene in Col-0 under water and drought condition. (**B**) The gene count of differentially expressed genes (P value ≤ 0.05 and FC ≥ 2) of WT-C/M1-C in leaf and root tissue. (C) the GO annotation of the differentially expressed genes in WT-C/M1-C in leaf and root tissue. Click here for file

Additional file 11 **Gene description of abiotic stress related genes in CAMTA1 dependent genes of leaf tissue.** List of CAMTA positively regulated genes related to various abiotic stress conditions in leaf tissue (LCDPRG) (work sheet 1). List of CAMTA negatively regulated genes related to various abiotic stress conditions in leaf tissue (LCDNRG) (work sheet 2). Percentage of CAMTA recognition motif (CGCG and CGTG) in various stress conditions (work sheet 3). Click here for file

Additional file 12 **Gene description of abiotic stress related genes in CAMTA1 dependent genes of root tissue.** List of CAMTA positively regulated genes related to various abiotic stress conditions in root tissue (RCDPRG) (work sheet 1). List of CAMTA negatively regulated genes related to various abiotic stress conditions in root tissue (RCDNRG) (work sheet 2). Percentage of CAMTA recognition motif (CGCG and CGTG) in various stress conditions (work sheet 3). Click here for file

Additional file 13 **Gene description of phytohormone associated genes in CAMTA1 dependent genes of leaf tissue.** List of phytohormones associated genes positively regulated by CAMTA in leaf tissue (LCDPRG) (work sheet 1). List of phytohormones associated genes negatively regulated by CAMTA in leaf tissue (LCDNRG) (work sheet 2). Click here for file

Additional file 14 **Gene description of phytohormone associated genes in CAMTA1 dependent genes of root tissue.** List of phytohormones associated genes positively regulated by CAMTA in root tissue (RCDPRG) (work sheet 1). List of phytohormones associated genes negatively regulated by CAMTA in root tissue (RCDNRG) (work sheet 2). Click here for file

Additional file 15 **Gene description of transcription factor in CAMTA1 dependent genes of leaf tissue.** List of TF positively regulated by CAMTA in leaf tissue (LCDPRG) (work sheet 1). List of TF negatively regulated by CAMTA in leaf tissue (LCDNRG) (work sheet). Click here for file

Additional file 16 **Gene description of transcription factor in CAMTA1 dependent genes of root tissue.** List of TF positively regulated by CAMTA in root tissue (RCDPRG) (work sheet 1). List of TF negatively regulated by CAMTA in leaf tissue (RCDNRG) (work sheet). Click here for file

Additional file 17 **The comparison of transcriptome of *camta1-2 *and *camta1-3. ***(**A**) The correlation graph between *camta1-2* and *camta1-3* gene expression profile from microarray data. The venn analysis shows unique and common significantly expressed genes in both mutants. (**B**) The Mapman analysis of *camta1-3.* Click here for file

Additional file 18 Primer sequence of genes used for validation by RT-PCR. Click here for file
