# Supplementary material for: CAMTA 1 regulates drought responses in Arabidopsis thaliana
Source: BMC Genomics. 2013 Apr 2;14:216. doi: 10.1186/1471-2164-14-216 (PMC3621073; doi:10.1186/1471-2164-14-216)
Supplement: Additional file 1 — Detailed information of ATCAMTA1-6 mutant with T-DNA insertion site. [file 1471-2164-14-216-S1.pdf]

Gene name	Locus	Germplams	Genotype	background	Polymorphism site
CAMTA 1	AT5G09410	SALK_108806, SALK_008187	Homozygous	Col-0	Exon
CAMTA 2	AT5G64220	SALK_014733	Homozygous	Col-0	Promoter
CAMTA 3	AT2G22300	SALK_001152	Homozygous	Col-0	Exon
CAMTA 4	AT1G67310	SALK_087870	Homozygous	Col-0	Exon
CAMTA 5	AT4G16150	SALK_120516	Homozygous	Col-0	Promoter
CAMTA 6	AT3G16940	SALK_078900	Homozygous	Col-0	Exon

Additional file 1
