# Supplementary figures and images for: CAMTA 1 regulates drought responses in Arabidopsis thaliana
Source: BMC Genomics. 2013 Apr 2;14:216. doi: 10.1186/1471-2164-14-216 (PMC3621073; doi:10.1186/1471-2164-14-216)

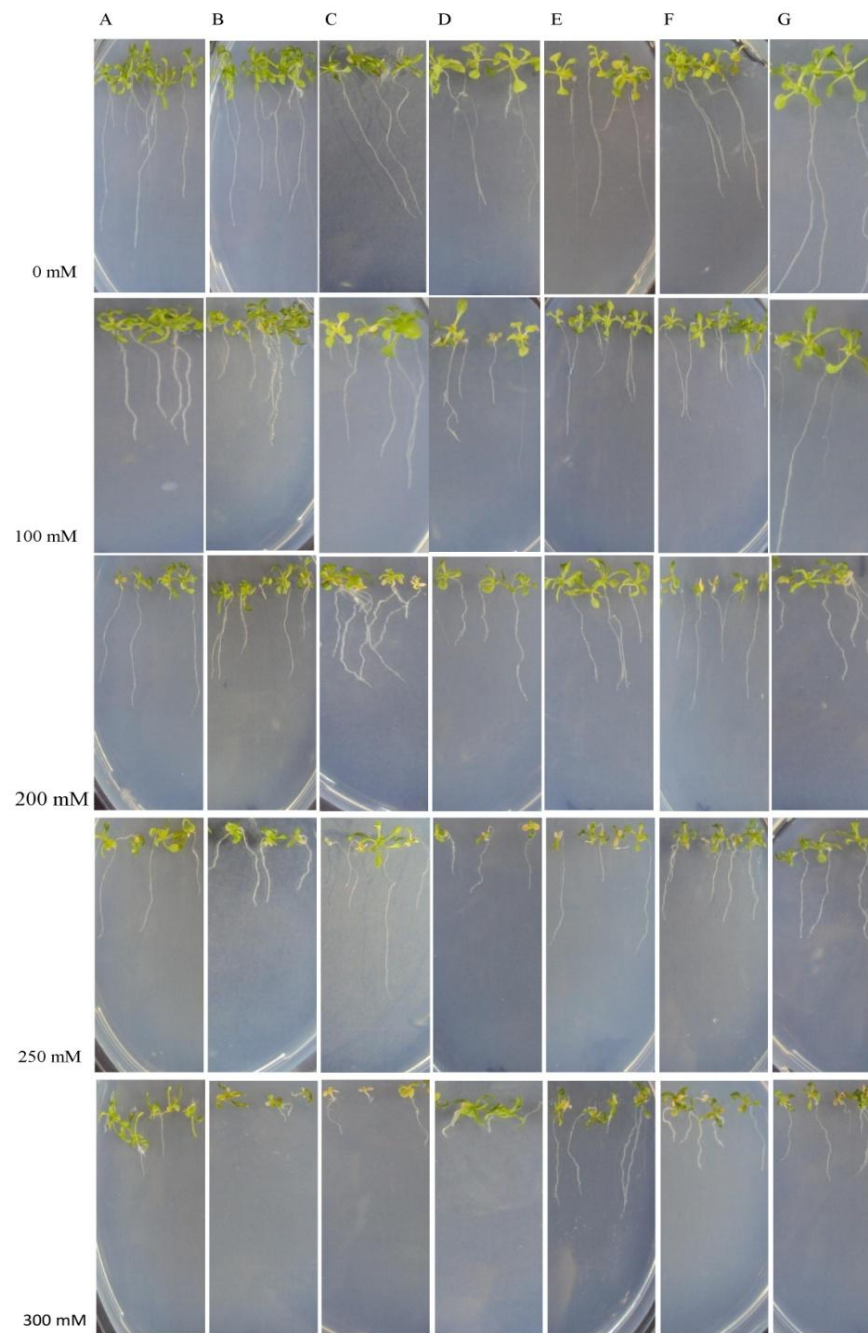

Supplement: Additional file 2 — Screening of different CAMTA mutant for drought tolerance. By using different mannitol concentration in MS media by root bending assay to show differential primary root growth of Col-0 and CAMTA mutant. (A) Col-0, (B) camta1, (C) camta2, (D) camta3, (E) camta4, (F) camta5, (G) camta6. [file 1471-2164-14-216-S2.pdf]
