# Supplementary material for: CAMTA 1 regulates drought responses in Arabidopsis thaliana
Source: BMC Genomics. 2013 Apr 2;14:216. doi: 10.1186/1471-2164-14-216 (PMC3621073; doi:10.1186/1471-2164-14-216)
Supplement: Additional file 3 — The phenotypic characterisation of camta1 mutant and Col-0 under drought stress and the relative expression of CAMTA1 in mutant and Col-0. Quantitative phenotypic measurements of Col-0, camta1-2 and camta1-3. The 3 weeks old plants were subjected to drought stress and after 14 days of stress the measurements have been recorded. Data are given as averages ± SD for 25 plants.The Expression of CAMTA1 gene in Col-0 and camta1-2 and camta1-3 by RT-PCR. [file 1471-2164-14-216-S3.pdf]

Parameters	Unit	Col-0	*camta1-2*	*camta1-3*
Number of rosette leaves	Count	15.22 ± 6.47	11.66 ± 4.63	10.01 ± 4.89
Leaf area	mm^2	29.06 ± 1.76	23.14 ± 2.93	24.27 ± 2.08
Rosette radius	mm	27.85 ± 2.19	16.1 ± 2.56	18.71 ± 4.19
Primary root length	mm	61.55 ± 5.69	38.28 ± 4.11	43.39 ± 2.70

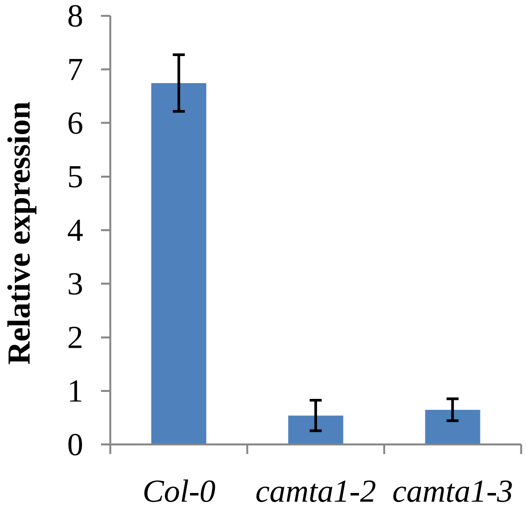

Additional file 3
