# Supplementary material for: CAMTA 1 regulates drought responses in Arabidopsis thaliana
Source: BMC Genomics. 2013 Apr 2;14:216. doi: 10.1186/1471-2164-14-216 (PMC3621073; doi:10.1186/1471-2164-14-216)
Supplement: Additional file 10 — The comparison between Col-0 and camta1-3 under water condition. (A) The relative expression of CAMTA1 gene in Col-0 under water and drought condition. (B) The gene count of differentially expressed genes (P value ≤ 0.05 and FC ≥ 2) of WT-C/M1-C in leaf and root tissue. (C) the GO annotation of the differentially expressed genes in WT-C/M1-C in leaf and root tissue. [file 1471-2164-14-216-S10.pdf]

A

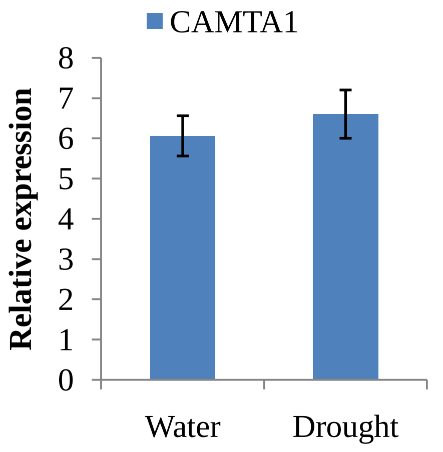

B

Condition	Up regulated	Down regulated
WT-C/M1-C (Leaf tissue)	209	169
WT-C/M1-C (Root tissue)	670	635

gene count of differentially expressed genes
(P value ≤ 0.05 and FC ≥ 2)

C

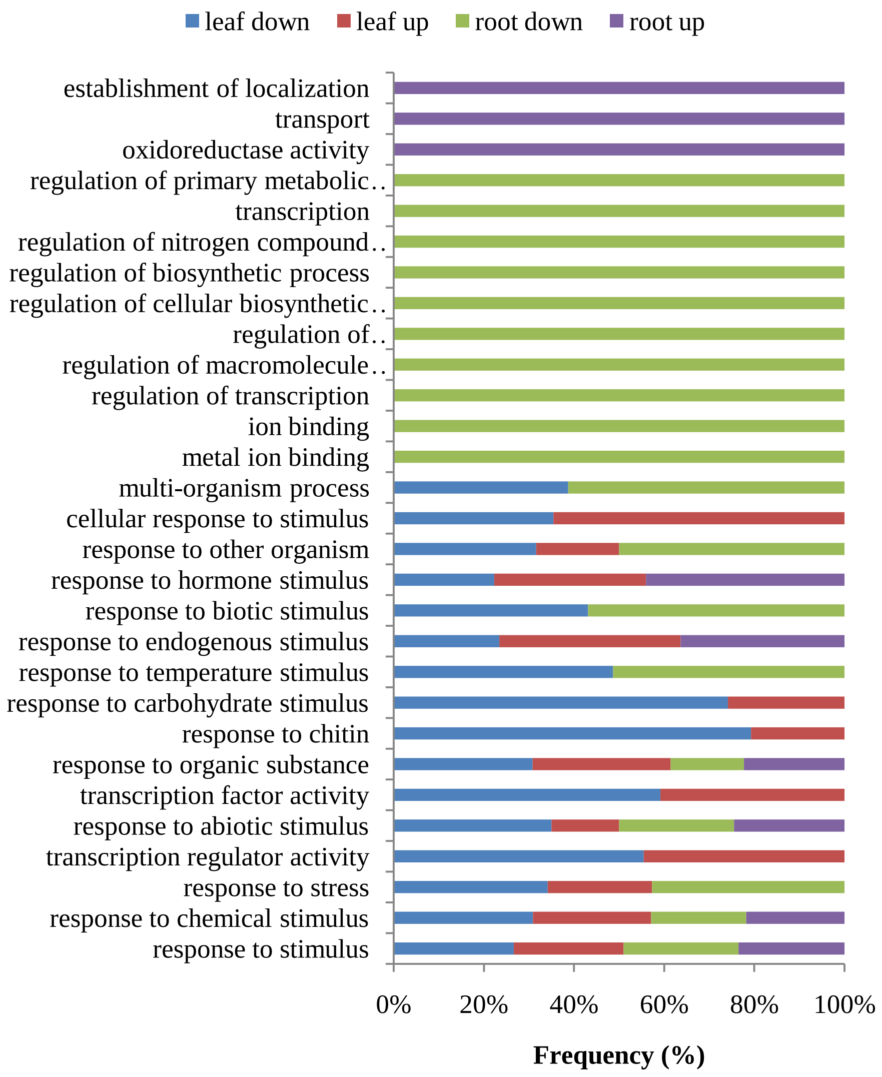

Additional file 10
