# Supplementary material for: CAMTA 1 regulates drought responses in Arabidopsis thaliana
Source: BMC Genomics. 2013 Apr 2;14:216. doi: 10.1186/1471-2164-14-216 (PMC3621073; doi:10.1186/1471-2164-14-216)
Supplement: Additional file 17 — The comparison of transcriptome of camta1-2 and camta1-3. (A) The correlation graph between camta1-2 and camta1-3 gene expression profile from microarray data. The venn analysis shows unique and common significantly expressed genes in both mutants. (B) The Mapman analysis of camta1-3. [file 1471-2164-14-216-S17.pdf]

A

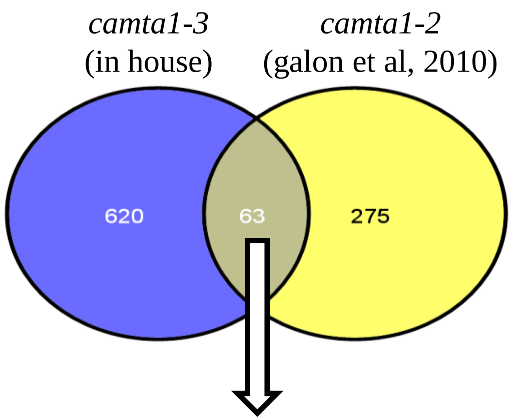

Probe Set ID	Locus Identifier	Gene Title	FC (*camta1-3*)	FC (*camta1-2*)
262212_at	AT1G74890	ARR15 (auxin response regulator15)	5.376499	2.723093
252415_at	AT3G47340	ASN1 (dark inducible 6)	4.958831	2.22684
247949_at	AT5G57220	cyp81f2 (cytochrome p450)	3.899617	9.57983
246108_at	AT5G28630	glycine-rich protein	2.751084	4.453687
259560_at	AT1G21270	WAK2 (wall-associated kinase)	2.639015	2.514027
246238_at	AT4G36670	mannitol transporter	2.276263	2.150989
250666_at	AT5G07100	WRKY26	2.271009	2.006943

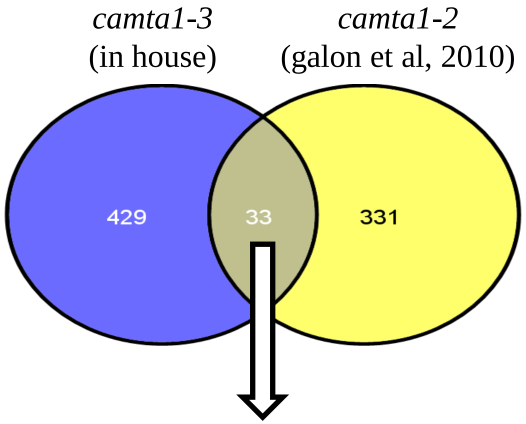

Probe Set ID	Locus Identifier	Gene Title	FC (*camta1-3*)	FC (*camta1-2*)
250207_at	AT5G13930	chalcone synthase	-2.82843	-2.36199
264931_at	AT1G60590	polygalacturonase	-2.75745	-2.46229
260955_at	AT1G06000	UDP-glucoronosyl	-2.55502	-2.77022
258321_at	AT3G22840	early light-inducable protein	-2.27626	-2.49666
265665_at	AT2G27420	cysteine proteinase	-2.04202	-6.56593
262382_at	AT1G72920	disease resistance protein	-2.65737	-4.16986
247543_at	AT5G61600	ethylene-responsive element-binding family protein	-2.56093	-2.51572

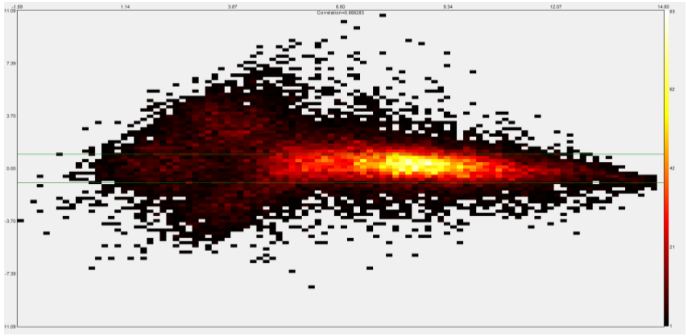

r = 0.86

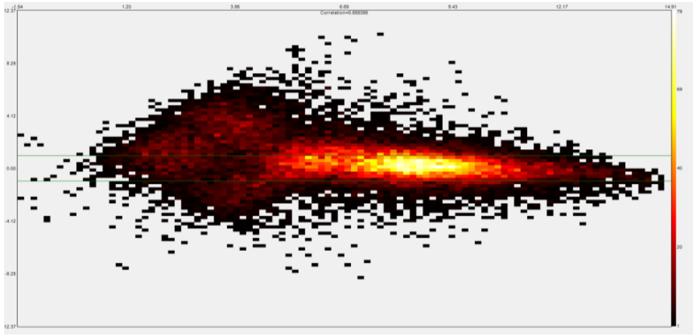

r = 0.85

B

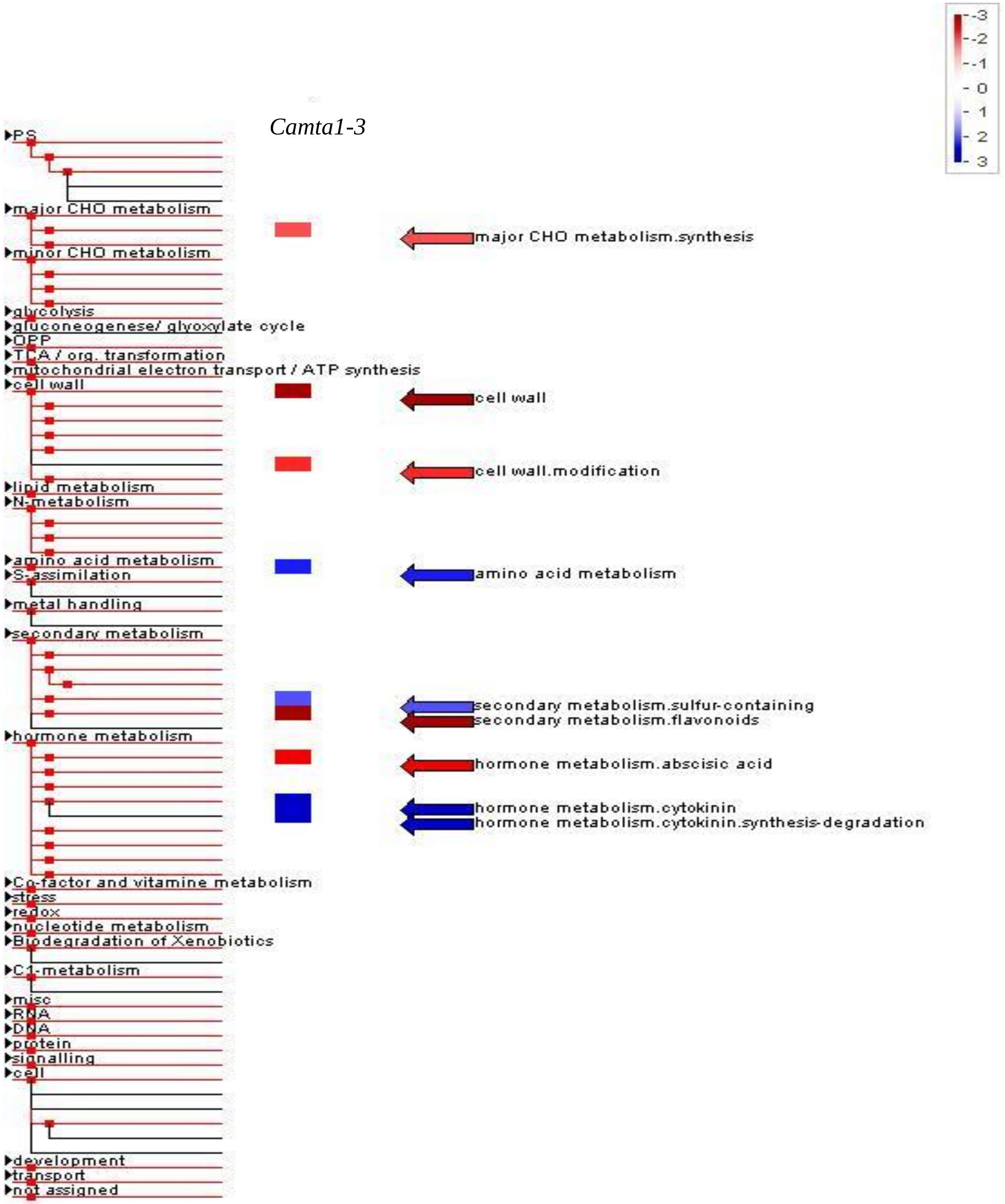

Additional file 17
