# Supplementary material for: CAMTA 1 regulates drought responses in Arabidopsis thaliana
Source: BMC Genomics. 2013 Apr 2;14:216. doi: 10.1186/1471-2164-14-216 (PMC3621073; doi:10.1186/1471-2164-14-216)
Supplement: Additional file 18 — Primer sequence of genes used for validation by RT-PCR. [file 1471-2164-14-216-S18.pdf]

Gene name	Forward Primer (5’-3’)	Reverse Primer (5’-3’)
glutathione S-transferase	GCGAGATACTACGCTACCAAGTTC	TCTAGAGACTTGCCCAAAAGGTT
anthocyanidin synthase	AGCCGTTGCCTGATATGGT	GCAAATGTCCTTGGAGGAAA
lipid transfer protein	GCACAGTGGCAAGTAGCTTGA	CACCACCCCACCCTTTGA
oxidoreductase	GGAGGATCGGGAGATTGC	AGCCGCGTTGAGGTCAGA
LEA protein	ATGCCGCCTAGCCTTATCA	CGCCTCGTTCCGAGTGA
dihydroflavonol 4-reductase	TCGGTCCATTCATCACAACGT	ATAGGAGAGAGCGCGGTGATA
ATCAMTA1	CCGGAAACAATACCGAACAG	TCCAACGCAAAATGATTTTCTC
Ubiquitin	GAAGCAGCTCGAGGATGGAA	CCACGGAGACGGAGGACA A

Additional file 18
